# Synthesis of Lactam-Bridged and Lipidated Cyclo-Peptides as Promising Anti-Phytopathogenic Agents

**DOI:** 10.3390/molecules25040811

**Published:** 2020-02-13

**Authors:** Aldrin V. Vasco, Martina Brode, Yanira Méndez, Oscar Valdés, Daniel G. Rivera, Ludger A. Wessjohann

**Affiliations:** 1 Department of Bioorganic Chemistry, Leibniz Institute of Plant Biochemistry, Weinberg 3, 06120 Halle (Saale), Germany; Aldrin.VascoVidal@ipb-halle.de (A.V.V.); martina.brode@ipb-halle.de (M.B.); Yanira.MendezGomez@ipb-halle.de (Y.M.); 2 Center for Natural Products Research, Faculty of Chemistry, University of Havana, Zapata y G, Havana 10400, Cuba; 3 Vicerrectoría de Investigación y Postgrado, Universidad Católica del Maule, Talca 3460000, Chile; ovaldes@ucm.cl

**Keywords:** peptide cyclization, antimicrobial peptides (AMPs), multicomponent reactions (MCRs), lipopeptides, fungicides, antimycotics, plant pathogens

## Abstract

Antimicrobial resistance to conventional antibiotics and the limited alternatives to combat plant-threatening pathogens are worldwide problems. Antibiotic lipopeptides exert remarkable membrane activity, which usually is not prone to fast resistance formation, and often show organism-type selectivity. Additional modes of action commonly complement the bioactivity profiles of such compounds. The present work describes a multicomponent-based methodology for the synthesis of cyclic polycationic lipopeptides with stabilized helical structures. The protocol comprises an on solid support Ugi-4-component macrocyclization in the presence of a lipidic isocyanide. Circular dichroism was employed to study the influence of both macrocyclization and lipidation on the amphiphilic helical structure in water and micellar media. First bioactivity studies against model phytopathogens demonstrated a positive effect of the lipidation on the antimicrobial activity.

## 1. Introduction

The continuous incidence of bacterial and fungal resistance has aroused great attention and re-sparked the interest in discovering or designing alternative antimicrobial substances that can be used for various applications including clinical uses as well as the preservation of food and dairy products [1,2,3]. Phytopathogenic microorganisms affect not only the yield of crop plants or stored foods (e.g., fruits) but also produce toxins, which have been described to be potentially harmful to the health of consumers and animals, including the induction of cancer, immunosuppression, and growth disorders [4,5,6]. Furthermore, the development of environmentally friendly and resistance-withstanding alternatives to the currently used pesticides remains one of the biggest challenges for plant scientists [7]. Polycationic antimicrobial peptides (AMPs) are essential components in the innate immune system of all multicellular organisms [8,9,10]. Even when the mechanisms of action of AMPs are not fully understood, in most cases their biological effects are believed to involve membrane disruption of the target cells by either acting through a detergent-like disruption or by the formation of transient transmembrane pores [11,12,13].

An increasing amount of articles describing the use of AMPs as antimycotic agents—including phytopathogenic fungi—has been published in the last few years [3,7,14,15,16,17,18]. Due to the eukaryotic nature of fungal cells, it is not an easy task to develop antifungal (antimycotic) drugs which do not exhibit lytic activity against plant and human cells [19]. Antimicrobial peptides, including antimicrobial biosurfactants, have been reported to act as both narrow and broad-spectrum agents against Gram-positive and Gram-negative bacteria, or fungi [20]. Ligation of AMPs to lipidic chains has proved effective for the design of broad-spectrum antifungal peptides with a low hemolytic activity, which can be designed to exhibit their bioactivity in a pH-dependent manner [8]. Lipidation of the peptide chain is broadly present in nature and it is a structural feature of several biosurfactants such as the Surfactin [21], Iturin [22], and Fengycin [23] families. These families of *Bacillus* lipopeptides have shown antagonistic activities towards a wide range of phytopathogens including fungi, bacteria and oomycetes [15]. Lipopeptides are produced non-ribosomally in bacteria, yeasts and fungi, and can act either over specific targets or by disrupting the cellular membrane. Subsequently, this type of antimicrobials has the advantage that it takes several hundred generations at low concentrations to develop bacterial resistance, a reason why they are widely considered as potential alternatives to the growing problem of resistance to conventional (protein targeting) antibiotics [3,15].

Our group took advantage of the high efficiency and atom economy of multicomponent reactions (MCRs) for macrocyclization and simultaneous peptide ligation and modification [24,25], e.g., for lipidation [26,27,28], glycosylation [29], pegylation and labeling [30]. We have shown how MCRs can be useful for the design of cyclic lipopeptide analogs of naturally-occurring biosurfactants and cytotoxins [31]. In a recent report, we developed a solid-supported Ugi-based stapling protocol for the convenient functionalization of short helical peptides [30]. Contemporary stapling protocols—mostly based on alkene metathesis [32,33], lactam bridge formation [34,35,36], Cu^I^-catalyzed alkyne-azide cycloaddition (click) [37], Lys-*N*^ε^- and Cys-*S*-arylations [38,39,40], and Cys-alkylation [41,42]—comprise the common stabilization methods for helical secondary structures by (side) chain-to-side chain tethering of two amino acids on the same face of an ideal helix, i.e., between amino acids at positions *i*→*i* + 4, *i*→*i* + 7, or *i*→*i* + 11 within the peptidic sequence. Likewise, the Ugi-based approach allows the helical stabilization by reacting side chains from Lys and Glu/Asp amino acids at *i*→*i* + 4 positions while incorporating the moiety arising from the isocyanide component as an additional functionalization at the resulting tertiary lactam bridge [30].

Herein we present the synthesis of two series of helical polycationic cyclic lipopeptides designed to possess a facial amphiphilic character. We envisioned that the introduction of a lipid moiety at the closing lactam bridge opposite to the polycationic face of the peptide—achievable through a Ugi-based multicomponent stapling approach—should stabilize the helical structure of these peptides and simultaneously enhance their amphiphilic character. To assess how these structural modifications can influence the bioactivity profile of polycationic lipopeptides, we sought to evaluate the activity of the compounds against three crop-affecting phytopathogens, which are also standard pathogens in industrial plant protection screenings.

## 2. Results and Discussion

To evaluate the suitability of the simultaneous macrocyclization and *N*-lipidation achievable by the Ugi-stapling methodology for the design of lactam-bridged *N*-lipidated peptides with antifungal activity, two sets of helical amphiphilic peptides were prepared. We aimed at evaluating how the lipidation at the lactam bridge affects the bioactivity of polycationic helical peptides as compared with their non-lipidated lactam-bridged analogs.

### 2.1. Synthesis of Peptides Based on the Ac-LAKLLKAKAKAD-NH_2_ Sequence

As a starting point for our study, we decided to design a peptide with 12 amino acids in its sequence—which is equivalent to three turns of an ideal α-helix—the facial amphiphilicity of which could arise from the distribution of cationic and lipophilic side chains at one vs. the other face of the helix, respectively. This design has previously proven effective to stabilize helical structures when Ugi-stapling between residues 5 and 9 is conducted [30]. Herein we intended to perform the cyclization at the *N*-terminus, envisioning that in this way the interaction of a lipid functionalization with the hydrophobic face of the helix would be higher and sterically less disturbing than when the stapling is situated in the middle of the sequence, resulting in a peptide with better hydrophobicity / amphiphilicity, which is important for membrane activity.

**Scheme 1:**
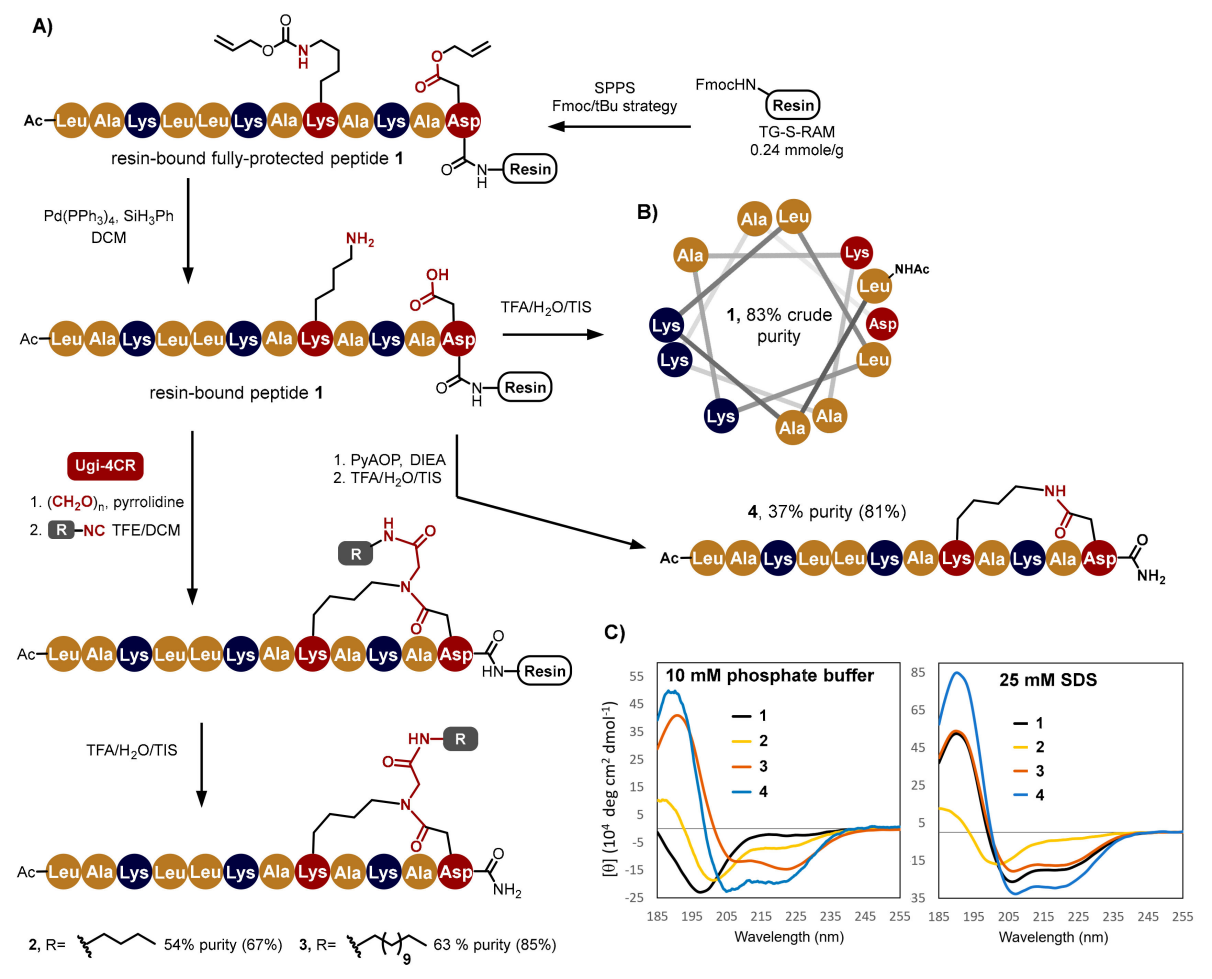
(**A**) Synthesis of polycationic dodecapeptides based on the peptide Ac-LAKLLKAKAKAD-NH_2_. In parenthesis are the yields of crude products; an analytical sample was purified to >93% purity; (**B**) Helical wheel representation of peptide **1** showing the disposition of the amino acids to be selectively modified (Lys8 and Asp12), located—under helix formation—opposite to the positively charged lysins at positions 3, 6 and 10; (**C**) Circular Dichroism (CD) spectra of the synthesized peptides in 10 mM phosphate buffer and 25 mM SDS aqueous solutions.

Resin-bound, fully-protected peptide **1** was prepared on solid support using a classical Fmoc/tBu strategy (Scheme 1A), with the sequential incorporation of the Fmoc-protected amino acids and final *N*-terminal acetylation. To achieve the cyclic peptides **2**, **3** and **4**, a third dimension of orthogonality was inserted by Alloc and Allyl ester-protected Lys and Asp at positions 8 and 12, respectively. This enables the selective deprotection of these side chains by treatment with tetrakis(triphenylphosphine)palladium(0) and phenylsilane under a stream of nitrogen, thus leading to the resin-bound peptide **1**. The unprotected side chains can then be employed in the desired on-resin macrocyclization protocol. In this way, cyclic peptide **4** was prepared from resin-bound peptide **1** by lactam-bridge formation in the presence of PyAOP as an activating agent. Even when a resin with relatively low loading was employed, 20% of dimerization product was observed in the crude peptide by UHPLC-MS analysis. The simultaneous cleavage from the resin and Boc/*t*Bu side chain deprotections are achieved by treating the resin-bound peptide with the mixture TFA/water/TIS.

Similar to our recently reported on-resin stapling protocol [30], the Ugi macrocyclization was conducted in two consecutive synthetic steps from resin-bound peptide **1**. The free amino group at Lys8 is converted to the corresponding imine by a transimination reaction with a mixture of pyrrolidine and paraformaldehyde. The macrocyclization is then accomplished by addition of the isocyanide component, which provides the desired functionalization at the *N*-position of the lactam bridge. Following this procedure, the synthesis of peptides **2** and **3** was conducted with the utilization of *n*-butyl and *n*-dodecyl isocyanide, respectively, in order to compare the influence of short and longer length aliphatic *N*-substitution on the biological activity. It should be noticed that no dimerization product was detected for any of the Ugi-cyclizations. The final yields after High Performance Liquid Chromatography (HPLC) purification can be considered as good, considering that the purity of the linear precursor resin-bound peptide **1** after Allyl/Alloc deprotection is around 83%. Full conversion of the linear peptide was achieved after 12 h, which is comparable to standard macrocyclization protocols [43,44].

### 2.2. Secondary Structure and Bioactivity

As depicted in the helical wheel representation of peptide **1** in Scheme 1B, an ideal helical conformation of this molecule shall present one face of the helix bearing three positively charged lysine side chains. We hypothesized that the cyclization of the peptide—known to induce the folding of peptides into helices—and the introduction of lipophilic substitutions opposite to the cationic side chains would fix the helical conformation and reinforce the facial amphiphilicity which could affect, eventually, the bioactivity.

The comparison of the CD spectra in phosphate buffer of peptides **1** and **4** evidences that the lactam bridging between residues 8 and 12 in peptide **4** leads to a transition from a random coil to a classical helical structure in water. Interestingly, compounds **2** and **3**—bearing an Ugi-derived *N*-substituted lactam bridge and therefore combining cyclization and lipophilic functionalization—show a very different conformational behavior. While cyclic peptide **3** occurs in an α-helical conformation as inferred from the CD minima at 207 and 222 nm and the maximum at 190 nm, compound **2** shows a CD spectrum with a tendency to 3_10_- helicity as evidenced in the lower intensity at 190 and 222 nm and the deep minimum around 200 nm as compared with **3**. This tendency towards 3_10_-helicity of Ugi-stapled peptides bearing three positive charges in close proximity has been observed previously by our group [27], and recent studies point out that it could be related to the occurrence of *s-cis*/*s-trans* isomerization only significantly happening in *N*-substituted lactam bridges (unpublished results). Altogether it seems that the Lys8-Asp12 cyclization contributes to nucleate helical conformations within this sequence, thereby ensuring the existence of three cationic lysines on one face of the molecule. While the non-functionalized lactam stapling ensures maximal helicity, the Ugi-derived *N*-substituted lactam bridge enables helix formation only when an increased amphiphilicity is achieved by insertion of a lipidic substituent. The CD spectra of the compounds in 25 mM sodium dodecyl sulfate (SDS)—commonly accepted to be a system mimicking the micellar environment of lipidic membranes [17]—shows an enhancement of helicity for peptides **3** and **4,** while the lower intensity of the minimum at 221 nm indicates a higher tendency to a random-coil structure for peptide **2** as compared with phosphate buffer. More conspicuous is the relatively high helicity of the linear analog **1**, which exhibits a helicity comparable to that of cyclic lipopeptide **3**. It must be noticed that the SDS model is very distant from the real cell membrane environment in fungi, and this structural characterization should only be used to estimate the capacity of the molecules to adopt amphiphilic structures in membrane-like environments which have been described to be of great importance for the membranolytic activity of several AMPs [11].

The activity of the synthesized compounds was tested against the phytopathogenic ascomycetes *Botrytis cinerea* Pers. (BC; grey mold pathogen on many crops, including strawberries and wine grapes), *Septoria tritici* Desm. (ST; causing septoria leaf blotch of wheat), and the oomycete *Phytophthora infestans* (Mont.) de Bary (PI; causal agent of the late blight disease in potato and tomato) (Table 1). While linear peptide **1** does not show any significant bioactivity, the lactam bridge cyclization in compound **4** results in an only moderate activity against PI. In the case of the Ugi-cyclized peptides, compound **2**—bearing an *n*-butyl *N*-substitution—shows a similar bioactivity as compound **4**, both with only moderate activity against the oomycete pathogen. A better bioactivity profile shows peptide **3**, whose *n*-dodecyl group through Ugi-stapling led to the best activity against ST and BC pathogens in this series of compounds.

Overall, there is no correlation between anti-phytopathogenic activity and the secondary structure within these molecules. However, there is indeed one between the activity and the presence of lipidation. Thus, while peptide **4** shows maximum helicity in both, phosphate buffer and micellar media, its activity is rather low and comparable to that of cyclic peptide **2**, which shows deviations from α-helical conformation in phosphate buffer and less helicity in SDS. Even when the peptides seem to be able to adopt helical structures in water or micellar medium—and therefore possess the facial amphiphilicity known to favor antimicrobial activity—the lipidation of the skeleton seems to be the most crucial structural modification for the bioactivity. The latter can be rationalized in two ways: i) the lipidation reinforces the amphiphilicity of the molecule by increasing the lipophilicity opposite to the polycationic face of the helix, and ii) this lipophilicity reinforces the interaction of the molecule with the hydrophobic membranes of the phytopathogens, leading to a better membranolytic activity.

### 2.3. Synthesis of Cyclic Lipopeptides Based on the (LKKL)_3_ Sequence

**Figure 1:**
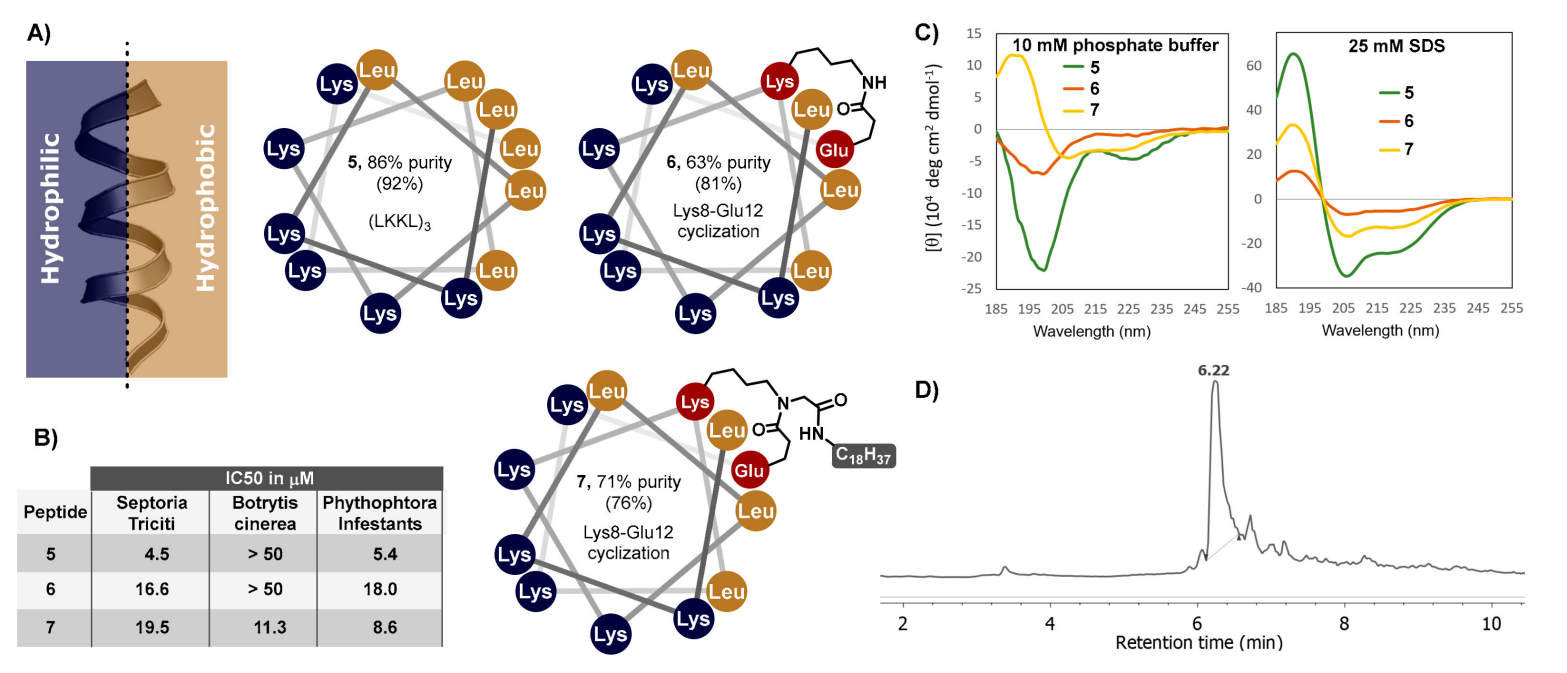
(**A**) Helical wheel representation of the structure of peptides **5-7** designed to possess an ideal amphiphilic structure. In parenthesis the yields of crude products; an analytical sample was purified to >93% purity; (**B**) Bioactivity of the compounds against model phytopathogens; (**C**) CD spectra for the peptides in 10 mM phosphate buffer and 25 mM SDS aqueous solutions; (**D**) UHPLC-MS chromatogram of crude peptide **7**.

To further assess the influence of the Ugi macrocyclizing lipidation over the antimicrobial activity of amphiphilic peptides, a second set of compounds was prepared following similar synthetic strategies as those described in Section 2.1. In this case, we chose to synthesize analogs of the sequence (LKKL)_3_ (peptide **6**) which has been described to possess potent antimicrobial bioactivity against drug-resistant biofilms of Gram-positive and Gram-negative bacteria, with low hemolytic activity at micromolar concentration [45]. Furthermore, for analogs of this compound it has been reported that an *N*-terminal lipidation is capable of broadening the antimicrobial spectrum towards human-associated pathogenic fungi [8]. As depicted in Figure 1A, peptide **5** presents an idealized amphiphilic helical conformation, in which half of the amino acids possess cationic side chains while the rest are of hydrophobic nature (Figure 1A).

Nevertheless, the helical conformation of these peptides is only evidenced when the CD spectra are recorded in SDS solution, while in phosphate buffer the CDs correspond to those of random-coil structures (Figure 1C). As in the previous examples, the cyclic analog **6** bearing a lactam-bridge was prepared for comparison purposes. For this peptide, macrocyclization does not guarantee or support helicity of the molecule in phosphate buffer, and only a moderate helicity in micellar media, as a comparison with the linear analog shows.

In contrast to macrocyclization only, simultaneous cyclization and insertion of an *n*-octadecyl moiety via Ugi-stapling improves helix induction in phosphate buffer as evidenced by the CD spectrum of peptide **7** (Figure 1C). In this case, a longer lipidic chain capable to contrast the higher net positive charge of the molecule was employed. As in peptide **3**, the introduction of the hydrophobic functionalization opposite to the polycationic face of the helix is expected to reinforce the facial amphiphilicity with the subsequent stabilization of the helical conformation. This stabilization could also be the result of intermolecular interactions due to the increased surfactant properties of the molecule when a lipidic chain is attached. This suspected intermolecular effect is corroborated by the fact that the micellar medium favors the helical conformations for the three compounds as shown in the CD spectra in Figure 1C-SDS. The Ugi macrocyclization for the synthesis of these cyclic lipopeptides is a very efficient synthetic approach as can be stated by the UHPLC-MS chromatogram of the crude lipopeptide after cleavage from the resin showing 71% purity (Figure 1D).

Among these three compounds, peptide **5** shows the highest activity against the phytopathogens ST and PI as shown in Figure 1B, and lower activity against BC. The conventional lactam-type cyclization of the skeleton as in compound **6** leads to a decrease of the bioactivity, with IC_50_ values being three times higher as for the linear analog. The latter is in correlation with the decrease in helicity in SDS solution observed in the CD spectrum, possibly due to some distortion due to intramolecular hydrogen-bonding interactions between the lactam bridge and one of the polycationic side chains. As compared with **6**, the insertion of the Ugi lipidation at the lactam bridge in **7** enhances the activity against PI slightly and extends the spectrum of activity to the BC pathogen, very likely due to the advent of biosurfactant properties absent in **5** and **6**.

**Figure 2:**
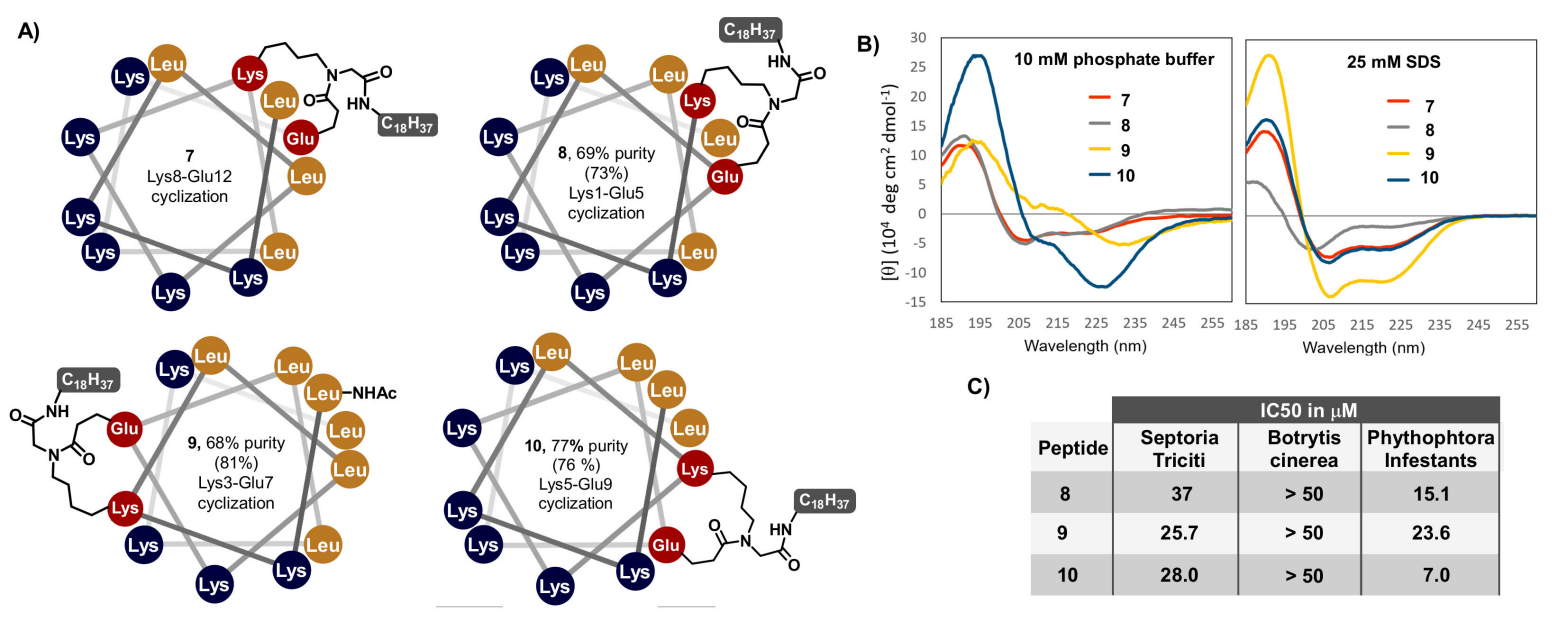
(**A**) Helical wheel representation of the structure of peptides **9-11.** In parenthesis the yields of crude products are given; an analytical sample was purified to >93% purity; (**B**) CD spectra for the peptides in 10 mM phosphate buffer and 25 mM SDS aqueous solutions, including the spectrum of **8** for comparison purposes;(**C**) Bioactivity of the compounds against model phytopathogens.

Commonly, macrocyclization of linear bioactives can significantly increase or decrease activity, depending on which conformational space is stabilized by the process, a beneficial or detrimental one, respectively. Thus, in order to learn if the cyclization site could have an influence over the helicity or the bioactivity, peptides **8**–**10** were prepared (Figure 2A). We hypothesized that the introduction of the C_18_ lipid tail as Ugi-derived lactam *N*-substituent at one or the other face of the helix could reinforce but also disrupt the amphiphilic character. The CD analysis in phosphate buffer of cyclic lipopeptides **7**–**10** shown in Figure 2B strongly supports our hypothesis. Thus, in stapled lipopeptides **7** and **8** the helix nucleation by Ugi cyclization at the *C*-(Lys8-Glu12) or *N*-terminus (Lys1-Glu5), respectively, reinforces the amphiphilic character by placing the long lipid tail opposite to the highly cationic Lys-rich face. As a result, **7** and **8** display typical α-helical CD spectra in phosphate buffer (Figure 2B) which corroborates the correlation between amphiphilicity and helicity in this class of structures. In contrast, peptides **9**—with the *N*-lipidation inserted into the polycationic face—and **10**—with the *N*-lipidation at the interface of the hydrophobic and hydrophilic side—show a deviation from helicity towards a β-sheet-like CD. However, these differences disappear when the CD spectra are recorded in 25 mM aqueous SDS solution and, surprisingly, while peptide **9** exerts the highest helicity, some 3_10_-helical behavior is observed for peptide **8**.

As compared with **7**, peptide **8** is less active against the three tested phytopathogens, indicating that not only the lipidation but also the directionality of the Ugi stapling is relevant for the bioactivity. Even when the last observation is in agreement with the diminished helicity of peptide **9** in SDS solution, the high helicity in micellar medium of peptide **9**—in which the ideal amphiphilicity is broken by the insertion of the Ugi lipidation—is not reflected in its activity against the model phytopathogens. Overall, the bioactive profile of peptides **8**–**10** is quite similar and none of them shows significant activity against BC. Broad-spectrum antimicrobial peptides with imperfectly amphipathic nature and low structural dependence on the bioactivity have been previously described by Wimley and co-workers who have supported the hypothesis that the interfacial activity could be the main determinant of the antimicrobial activity of AMPs, governed by physico-chemical balance rather than the spatial arrangement of the hydrophobic and polar groups [20,46].

In summary, we have demonstrated how the Ugi-stapling methodology can be useful for the preparation of two series of antimicrobial macrocyclic lipopeptides in which the *N*-lipidation is inserted at the cyclizing lactam bridge. Compared with state-of-the-art cyclization methodologies, the high atom economy of this multicomponent cyclization stapling approach allows the uncomplicated and highly efficient access to helical peptides designed to possess a high facial amphiphilicity by the combination of polycationic side chains and Ugi-derived hydrophobic *N*-substitution. Furthermore, such a cyclo-lipidation methodology could be useful for the future design of helical AMPs with better pharmacological profiles, e.g., better proteolytic stability. The recorded CD spectra show a high propensity of the lipopeptides to stabilize helical structures in aqueous SDS solution, even when the *N*-substitution is situated at an unfavorable position. As compared with the non-lipidated analogs, the Ugi-derived lipidation of peptide **1** leads to increased bioactivity profiles against two model phytopathogenic fungi and one oomycete. In the case of the analogs of the (LKKL)_3_ sequence (peptide **5**), the *C*-terminal cyclo-lipidation improves the bioactive profile with a significant enhancement in the bioactivity against *Botrytis cinerea*. Although no mode of action study was conducted, the retention of activity in peptides where the lipidation is inserted at a position disfavourable for good amphiphilicity can be seen as indicative of a non-specific disruption of the cell membrane due to lipid-enabled interfacial activity of the peptides. Overall we have shown a rapid, easy to perform and simultaneous macrocyclization-lipidation method which will allow researchers to produce antifungal macrocycle-stabilized lipopeptides and stabilized helix-peptides. With some SAR effort it should be possible to generate compounds in the 1 µM activity range, commonly seen as best activity value obtainable for AMPs with physicochemical (membrane) mode of action.

## 3. Materials and Methods

### 3.1. Materials and Equipment

All materials were purchased from Merck KGaA (Darmstadt, Germany), Carl Roth GmbH&Co. KG (Karlsruhe, Germany), VWR International GmbH (Darmstadt, Germany), Carbolution Chemicals GmbH (St. Ingbert, Germany), and Iris Biotech GmbH (Marktredwitz, Germany) and were used as received, unless otherwise noted. Analytical HPLC of peptides **1**–**3** and **7**–**10** was performed on a Waters Acquity UHPLC BEH C18 column (1.7 μm, 2.1 mm × 50 mm) using a Waters Acquity UHPLC system coupled to an LCQ Deca XP MAX (Thermo Scientific) mass spectrometer and the ESI IT mass spectra were recorded with a 4.0 kV spray voltage; sheath gas nitrogen; capillary temperature, 275 °C; capillary voltage, 30 V. The column was maintained at 40 °C. Alternatively, analytical HPLC of compounds **4**–**6** was conducted with the same column in a Waters Acquity UHPLC system equipped with a PDA and a QDa detector. In this case, UV spectra from 200–400 nm were recorded and the ESI mass spectra were recorded with a 1.5 kV spray voltage. Unless otherwise stated, a linear gradient from 5% to 90% of solvent B (0.1% (*v*/*v*) formic acid (FA) in acetonitrile) in solvent A (0.1% (*v*/*v*) formic acid (FA) in water) over 10 min at a flow rate of 0.15 mL min^−1^ was used. The mass spectra were evaluated by the Thermo software Xcalibur 2.0.7 and Empower 3 software. Preparative RP-HPLC of crude peptides was carried out with an Agilent 1260 Infinity series system equipped with two preparative pumps and one analytical quaternary pump, coupled to a MWD detector and an Agilent 6120 Quadrupole LC/MS detector using API-ES as ion source. Reverse phase YMC-ODS-A column (150 × 4.6 mm I.D., 5 μm particle size) and YMC-ODS-A (150 × 20 mm I.D., 5 μm particle size) columns were used for analytical and preparative scale respectively.

A TripleToF 6600-1 mass spectrometer (Sciex) was used for high-resolution mass spectrometry, which was equipped with an ESI-DuoSpray-Ion-Source (it operated in positive ion mode) and was controlled by Analyst 1.7.1 TF software (Sciex). The ESI source operation parameters were as follows: ion spray voltage: 5500 V, nebulizing gas: 60 p.s.i., source temperature: 450 °C, drying gas: 70 p.s.i., curtain gas: 35 p.s.i. Data acquisition was performed in the MS1-ToF mode, scanned from 100 to 1500 Da with an accumulation time of 50 ms.

Circular dichroism spectra were recorded on a Jasco J-815 spectropolarimeter equipped with a temperature controller at 20 °C. A total of 16 accumulations from 260 to185 nm at 50 nm/sec using 1 mm cuvettes were performed.

Additional information such as compound spectra and detailed bioactivity data are provided in the Supplementary Materials.

### 3.2. General Procedures

#### 3.2.1. Solid Phase Peptide Synthesis

Coupling reactions were carried out automatically on an INTAVIS ResPepSL automated peptide synthesizer by a stepwise Fmoc/tBu strategy using a 5-fold excess of amino acid and PyBop and a 10-fold excess of NMM at R.T. for 15 min. Fmoc deprotection was achieved by treatment with 20% piperidine in *N*,*N*-dimethylformamide (DMF) solution. Washing steps, as well as peptide coupling reactions, were conducted using DMF as a solvent. A 50 µmol scale on TG-S-RAM (Iris Biotech) resin (217 mg, 0.23 mmol/mg) was utilized. Before bringing it into the synthesizer, the resin was swelled for 20 min in dichloromethane.

#### 3.2.2. *N*-Terminus Acetylation

After the coupling of all amino acids, the resin is manually washed with DCM (3 × 1 min) and DMF (2 × 1 min) and reacted with 10 eq. of Ac_2_O/DIEA in 2 mL of DMF for 30 min [47]. The completeness of the reaction was confirmed through the Kaiser test [48] and the resin was manually washed with DCM (3 × 1 min).

#### 3.2.3. Alloc/Allyl Ester Removal

The resin was washed with dry dichloromethane (2 × 2 min) under a stream of nitrogen. A solution of phenylsilane (20 eq.) in dry dichloromethane and tetrakis(triphenylphosphine) palladium(0) (0.2 eq.) were added to the resin under a continuous stream of nitrogen. The mixture was stirred or shaken in the dark for 10 min, and the procedure was repeated once more. [49] Finally, the resin was washed with 0.5% of sodium diethyldithiocarbamate trihydrate in DMF (5 × 2 min) and DCM (2 × 2 min).

#### 3.2.4. Aminocatalysis-Mediated Ugi-4C Cyclization

The side chain deprotected resin-bound peptide was washed with THF (4×1 min) and treated with a suspension of paraformaldehyde (4 eq.) and pyrrolidine (4 eq.) in THF/MeOH (1:1) for 30 min. The excess of reagents was removed by washing the beads with THF (4 × 1min). The resin was then washed with DCM (4 × 1min) and DCM/TFE 1:1 solution (2 × 1min). A solution of the isocyanide (4 eq.) in 2 mL DCM/TFE 1:1 (or THF:MeOH 1:1 for relative polar isocyanides) was added to the resin and the suspension was stirred overnight (18 h) [30]. Completion was evaluated by ESI-MS or RP-HPLC monitoring after mini-cleavages. Afterwards, the resin was washed with DCM (3×1 min) and DMF (2 × 1 min). Finally, the resin was washed with DCM (5 × 2 min) and Et_2_O (3 × 1 min).

#### 3.2.5. Cyclization by Peptide Coupling

PyAOP (4 equiv.) and DIPEA (6 equiv.) were dissolved in DMF and the solution was added to the resin-bound peptide similar to the described protocol from Amblard et al. for phosphonium-based activation [47]. The suspension was stirred for 12 h and then the resin was washed with DCM (3 × 1 min) and DMF (2 × 1 min).

#### 3.2.6. Test Cleavage (Mini-Cleavage)

To evaluate the completion of the reaction, a small amount of the dry resin (less than 10 mg) was transferred to an Eppendorf vial and 400 µL of the cleavage cocktail (TFA/TIS/H_2_O 95:2.5:2.5) was added. The suspension was gently agitated during 45 min and afterwards the cleavage cocktail was concentrated under N_2_ flow and the remaining oil was precipitated by addition of 500 µL diethyl ether, similar to the protocol described by Spring and co-workers [50]. The suspension was centrifuged for 5 min after which the solid and liquid phases were separated by settling. The precipitated peptide was dissolved in a mixture ACN/H_2_O in order to be analyzed by HPLC.

#### 3.2.7. Cleavage

The resin was treated with the cocktail TFA/TIS/H_2_O (95:2.5:2.5). The peptide was precipitated from cold diethyl ether, then taken up in 1:2 ACN/H_2_O and lyophilized [47].

### 3.3. Synthesis

*Resin-bound Ac-LAKLLKAKAKAD* (resin-bound peptide **1**) Resin-bound peptide Ac-Leu-Ala-Lys(Boc)-Leu-Leu-Lys(Boc)-Ala-Lys-Ala-Lys(Boc)-Ala-Asp was synthesized in 50 µmol scale according to the protocols described [30]. The purity of the resin-bound peptide was evaluated through test cleavages as described above. Analytical UHPLC: *R*_t_ 4.54 min, >83% purity (crude). Analytical UHPLC: *R*_t_ 2.86 min, >98% purity; ESI-HRMS, calcd. for C_60_H_114_N_17_O_15_^3+^: 437.6227 [M + 3H]^3+^; Found: *m*/*z* 437.6215 [M + 3H]^3+^.

*Ac-(N-(2-(n-butylamino)-2-oxoethyl)-(cyclo-8,12))-[LAKLLKAKAKAD]-NH_2_* (**2**) The title compound was produced by on-resin cyclization in a 50 µmol scale from resin-bound peptide **1** according to the aminocatalysis-mediated Ugi macrocyclization protocol in the presence of *n*-butylisocyanide. Cleavage with TFA/H_2_O/TIS 95:2.5:2.5 for 2 h afforded the crude peptide (47.2 mg, 67% crude yield, 54% purity) as an amorphous white solid. An analytical sample was purified by semipreparative RP-HPLC for bioassays and CD. Analytical UHPLC: *R*_t_ 6.19 min, >94% purity. ESI-HRMS, calcd. for C_66_H_123_N_18_O_15_^3+^: 469.3139 [M + 3H]^3+^ Found: *m*/*z* 469.3145 [M + 3H]^3+^.

*Ac-(N-(2-(n-dodecylamino)-2-oxoethyl)-(cyclo-8,12))-[LAKLLKAKAKAD]-NH_2_* (**3**) The title compound was produced by on-resin cyclization in a 50 µmol scale from resin-bound peptide **1** according to the aminocatalysis-mediated Ugi macrocyclization protocol in the presence of *n*-dodecylisocyanide. Cleavage with TFA/H_2_O/TIS 95:2.5:2.5 for 2 h afforded the crude peptide (64.3 mg, 85% crude yield, 63% purity) as an amorphous white solid. An analytical sample was purified by semipreparative RP-HPLC for bioassays and CD. Analytical UHPLC: *R*_t_ 8.17 min, >91% purity. ESI-HRMS, calcd. for C_74_H_139_N_18_O_15_^3+^: 506.6889 [M + 3H]^3+^ Found: *m*/*z* 506.6874 [M + 3H]^3+^.

*Ac-(cyclo-8,12))-[LAKLLKAKAKAD]-NH_2_* (**4**) The title compound was produced by on-resin cyclization in a 50 µmol scale from resin-bound peptide **1** according to the cyclization by peptide coupling protocol. Cleavage with TFA/H_2_O/TIS 95:2.5:2.5 for 2 h afforded the crude peptide (48.9 mg, 81% crude yield, 37% purity) as an amorphous white solid. An analytical sample was purified by semipreparative RP-HPLC for bioassays and CD. Analytical UHPLC: *R*_t_ 3.92 min, >89% purity. ESI-HRMS, calcd. for C_60_H_112_N_17_O_14_^3+^: 431.6192 [M + 3H]^3+^ Found: *m/z* 431.6180 [M + 3H]^3+^.

*Ac-LKKLLKKLLKKL* (**5**). The title compound was synthesized in 50 µmol according to the general procedure. The resulting product is cleaved from the resin to obtain after lyophilization the crude peptide (69.1 mg, 92% crude yield, 86% purity) as an amorphous white solid. An analytical sample was purified by semipreparative RP-HPLC for bioassays and CD. Analytical UHPLC: *R*_t_ 2.89 min, >98% purity; ESI-HRMS, calcd. for C_74_H_147_N_19_O_13_^4+^: 377,5357 [M + 4H]^4+^; Found: *m*/*z* 377,5349 [M + 4H]^4+^.

*Ac-(cyclo-8,12))-[LKKLLKKKLKKE]-NH_2_* (**6**) The title compound was produced by on-resin cyclization in a 50 µmol scale from resin-bound peptide Ac-*LKKLLKKKLKKE-(TG-S-RAM)* (all Lysines except Lys8 protected as *Boc*) according to the cyclization by peptide coupling protocol. Cleavage with TFA/H_2_O/TIS 95:2.5:2.5 for 2 h afforded the crude peptide (61.5 mg, 81% crude yield, 63% purity) as an amorphous white solid. An analytical sample was purified by semipreparative RP-HPLC for bioassays and CD. Analytical UHPLC: *R*_t_ 2.36 min, >93% purity. ESI-HRMS, calcd. for C_73_H_142_N_20_O_14_^4+^: 380.7754 [M + 4H]^4+^ Found: *m*/*z* 380.7748 [M + 4H]^4+^.

*Ac-(N-(2-(n-octadecylamino)-2-oxoethyl)-(cyclo-8,12))-[LKKLLKKKLKKE]-NH_2_* (**7**) The title compound was produced by on-resin cyclization in a 50 µmol scale from resin-bound peptide Ac-*LKKLLKKKLKKE-(TG-S-RAM)* (all Lysines except Lys8 protected as *Boc*) according to the aminocatalysis-mediated Ugi macrocyclization protocol in the presence of *n*-octadecylisocyanide. Cleavage with TFA/H_2_O/TIS 95:2.5:2.5 for 2 h afforded the crude peptide (68.1 mg, 76% crude yield, 71% purity) as an amorphous white solid. An analytical sample was purified by semipreparative RP-HPLC for bioassays and CD. Analytical UHPLC: *R*_t_ 6.61 min, >93% purity. ESI-HRMS, calcd. for C_91_H_180_N_21_O_14_^5+^: 358.2804 [M + 5H]^5+^ Found: *m*/*z* 358.2780 [M + 5H]^5+^.

*Ac-(N-(2-(n-octadecylamino)-2-oxoethyl)-(cyclo-1,5))-[KKKLEKKLLKKL]-NH_2_* (**8**) The title compound was produced by on-resin cyclization in a 50 µmol scale from resin-bound peptide Ac- *KKKLEKKLLKK-(TG-S-RAM)* (all Lysines except Lys1 protected as *Boc*) according to the aminocatalysis-mediated Ugi macrocyclization protocol in the presence of *n*-octadecylisocyanide. Cleavage with TFA/H_2_O/TIS 95:2.5:2.5 for 2 h afforded the crude peptide (65.2 mg, 73% crude yield, 69% purity) as an amorphous white solid. An analytical sample was purified by semipreparative RP-HPLC for bioassays and CD. Analytical UHPLC: *R*_t_ 6.48 min, >89% purity. ESI-HRMS, calcd. for C_91_H_180_N_21_O_14_^5+^: 358.2804 [M + 5H]^5+^ Found: *m*/*z* 358.2777 [M + 5H]^5+^.

*Ac-(N-(2-(n-octadecylamino)-2-oxoethyl)-(cyclo-3,7))-[LKKLLKELLKKL]-NH_2_* (**9**) The title compound was produced by on-resin cyclization in a 50 µmol scale from resin-bound peptide Ac- *LKKLLKELLKKL-(TG-S-RAM)* (all Lysines except Lys3 protected as *Boc*) according to the aminocatalysis-mediated Ugi macrocyclization protocol in the presence of *n*-octadecylisocyanide. Cleavage with TFA/H_2_O/TIS 95:2.5:2.5 for 2 h afforded the crude peptide (72.4 mg, 81% crude yield, 68% purity) as an amorphous white solid. An analytical sample was purified by semipreparative RP-HPLC for bioassays and CD. Analytical UHPLC: *R*_t_ 9.41 min, >90% purity. ESI-HRMS, calcd. for C_93_H_179_N_19_O_15_^4+^: 450.5957 [M + 4H]^4+^ Found: *m*/*z* 450.5929 [M + 4H]^4+^.

*Ac-(N-(2-(n-octadecylamino)-2-oxoethyl)-(cyclo-5,9))-[LKKLKKKLEKKL]-NH_2_* (**10**) The title compound was produced by on-resin cyclization in a 50 µmol scale from resin-bound peptide Ac- *LKKLKKKLEKKL-(TG-S-RAM)* (all Lysines except Lys5 protected as *Boc*) according to the aminocatalysis-mediated Ugi macrocyclization protocol in the presence of *n*-octadecylisocyanide. Cleavage with TFA/H_2_O/TIS 95:2.5:2.5 for 2 h afforded the crude peptide (66.8 mg, 75% crude yield, 77% purity) as an amorphous white solid. An analytical sample was purified by semipreparative RP-HPLC for bioassays and CD. Analytical UHPLC: *R*_t_ 6.67 min, >91% purity. ESI-HRMS, calcd. for C_91_H_180_N_21_O_14_^4+^: 358.2804 [M + 5H]^5+^ Found: *m*/*z* 358.2780 [M + 5H]^5+^.

### 3.4. Anti-Phytopathogenic Bioassays

The compounds were tested in 96-well microtiter plate assays against the phytopathogenic ascomycetes *Botrytis cinerea* Pers. and *Septoria tritici* Desm. and the oomycete *Phytophthora infestans* (Mont.) de Bary according to the monitoring methods approved by the fungicide resistance action committee (FRAC) [51,52,53] with minor modifications as described by Otto et al. [54]. Compounds were examined at 125, 42, 14, 4.6 and 1.5 µM and when necessary additional experiments were performed with higher dilution steps. The mother solution solvent DMSO was used as negative control (max. concentration 2.5%), while Epoxiconazole and Terbinafine served as positive controls. Seven days after inoculation, pathogen growth was evaluated by measurement of the optical density (OD) at λ 405 nm with a TecanSpark microplate reader. Each experiment was carried out in triplicate. IC_50_-values were calculated from dose−response curves on the basis of a linear regression.

## Figures and Tables

**Table 1:** Half maximal inhibitory concentration (in µmol/L) of peptides **2**–**4** against model phytopathogens. (For more, detailed data see Supplementary Material)

Peptide	*Septoria Tritici*	*Botrytis Cinerea*	*Phytophthora Infestans*
**1**	>50	>50	>50
**2**	>50	>50	26.2
**3**	5.9	9.8	14.3
**4**	>50	>50	20.9

## Supplementary Materials

The following are available online, Figure S1–S11: UHPLC-MS chromatograms and HRMS spectra of peptides **1**–**11**. Figure S12–S14: Inhibition of phytopathogenic growth by the macrocyclic lipopeptides.
