# Supplementary material for: Synthesis of Lactam-Bridged and Lipidated Cyclo-Peptides as Promising Anti-Phytopathogenic Agents
Source: Molecules. 2020 Feb 13;25(4):811. doi: 10.3390/molecules25040811 (PMC7070897; doi:10.3390/molecules25040811)
Supplement: Supplementary file 1 [file molecules-25-00811-s001.pdf]

Supporting Information

Synthesis of lactam-bridged and lipidated cyclo-peptides as promising anti-phytopathogenic agents

Aldrin V. Vasco,[1] Martina Brode,[1] Yanira Méndez,[1,2] Oscar Valdés,[3] Daniel G. Rivera,[1,2,*] and Ludger A. Wessjohann[1,*]

[1] Department of Bioorganic Chemistry, Leibniz Institute of Plant Biochemistry, Weinberg 3, 06120 Halle (Saale), Germany
[2] Center for Natural Products Research, Faculty of Chemistry, University of Havana, Zapata y G, Havana 10400, Cuba
[3] Vicerrectoría de Investigación y Postgrado, Universidad Católica del Maule, Talca 3460000, Chile

*Correspondence: dgr@fq.uh.cu; wessjohann@ipb-halle.de (ORCID 0000-0003-2060-8235)

Table of contents

Figure S1. Structure, UHPLC-MS trace ESI-HRMS spectrum of crude peptide **1** S2

Figure S2. Structure, UHPLC-MS trace and ESI-HRMS spectrum of pure peptide **2** S3

Figure S3. Structure, UHPLC-MS trace and ESI-HRMS spectrum of pure peptide **3** S4

Figure S4. Structure, UHPLC trace at 200 nm and ESI-HRMS spectrum of pure peptide **4** S5

Figure S5. Structure, UHPLC trace at 200 nm and ESI-HRMS spectrum of crude peptide **5** S6

Figure S6. Structure, UHPLC trace at 200 nm and ESI-HRMS spectrum of pure peptide **6** S7

Figure S7. Structure, UHPLC-MS trace and ESI-HRMS spectrum of pure peptide **7** S8

Figure S8. Structure, UHPLC-MS trace and ESI-HRMS spectrum of pure peptide **8** S9

Figure S9. Structure, UHPLC-MS trace and ESI-HRMS spectrum of pure peptide **9** S10

Figure S10. Structure, UHPLC-MS trace and ESI-HRMS spectrum of pure peptide **10** S11

Figure S11. Inhibitory activity of the compounds against *Septoria tritici* pathogen S12

Figure S12. Inhibitory activity of the compounds against *Botrytis cinerea* pathogen S13

Figure S13. Inhibitory activity of the compounds against *Phytophthora infestans* pathogen S14

Synthesis of isocyanides S14

A)

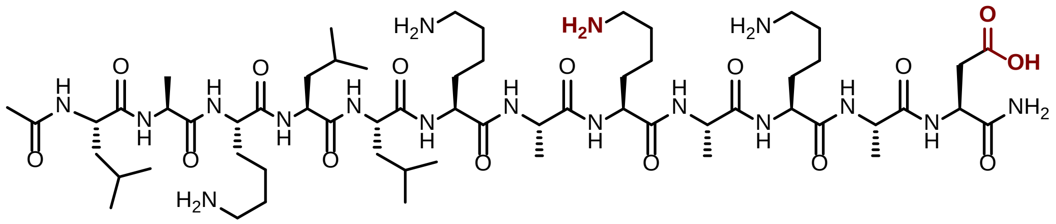

Chemical Formula: $C_{60}H_{111}N_{17}O_{15}$
Exact Mass: 1309.8446

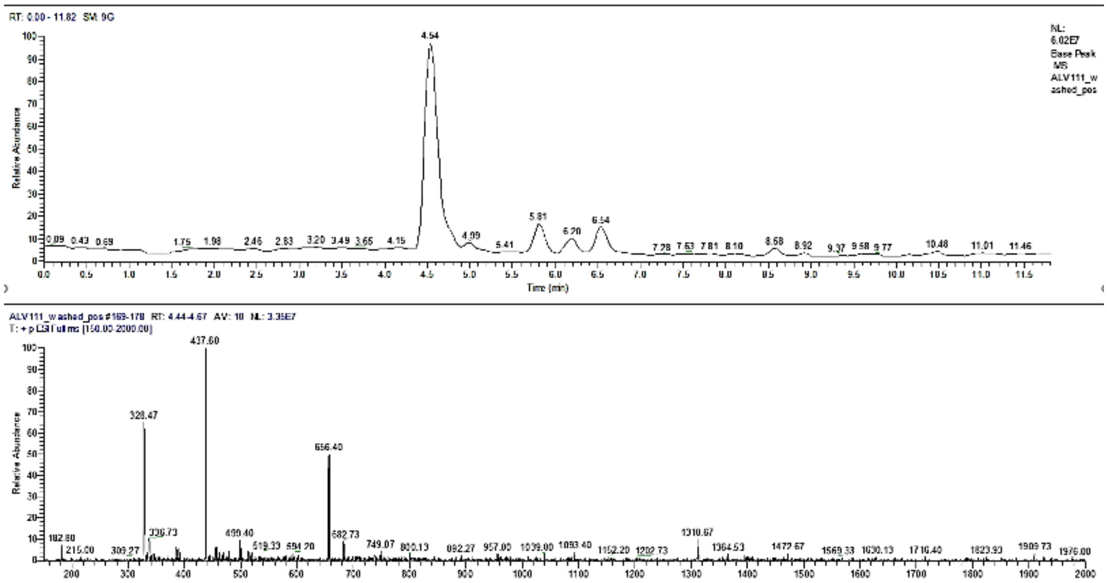

B)
C)

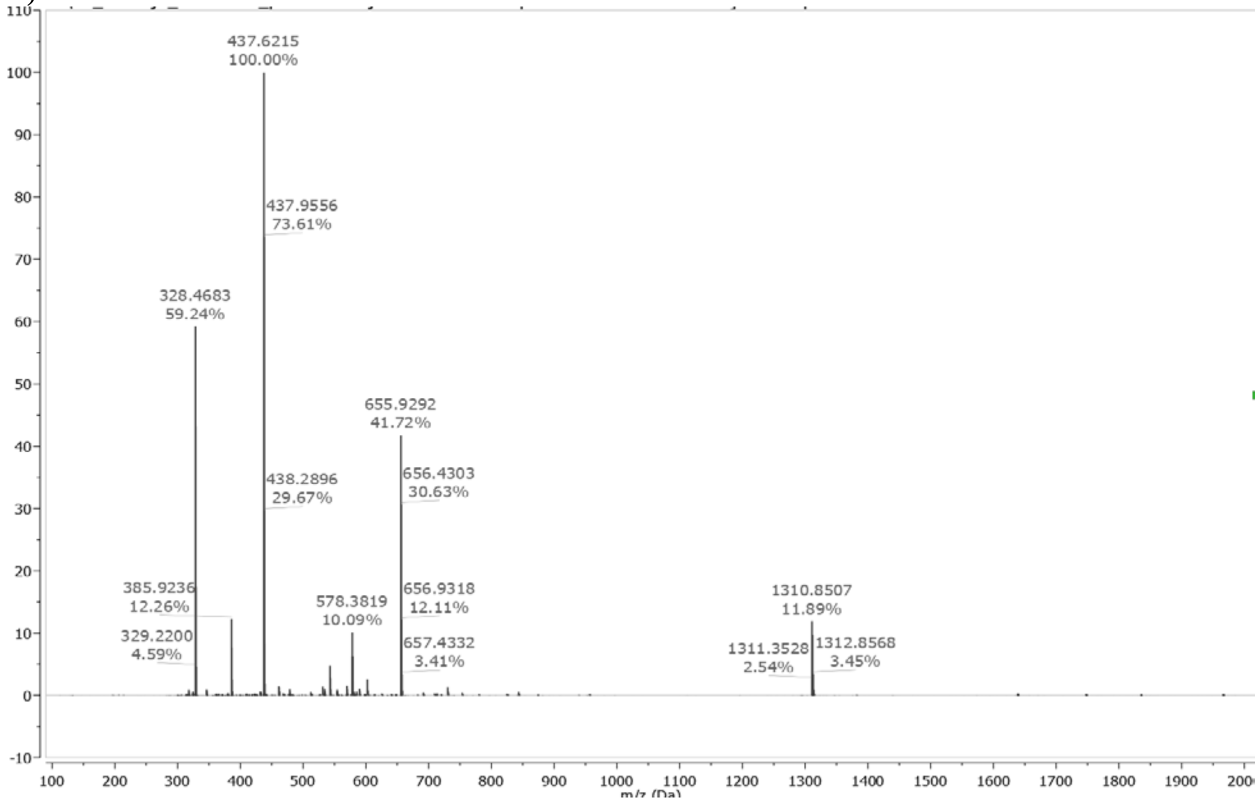

Figure S1. A) Structure, B) UHPLC-MS trace C) ESI-HRMS spectrum of crude peptide **1**

A)

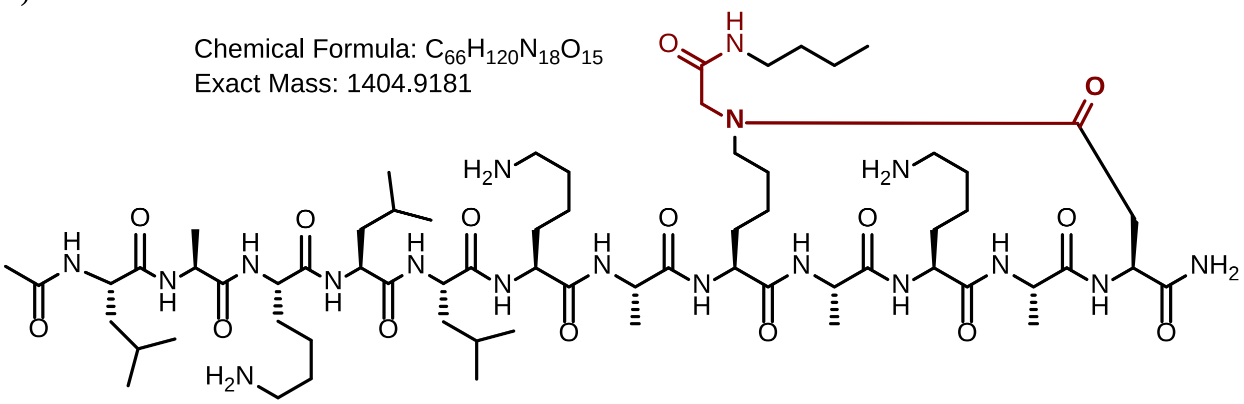

B)

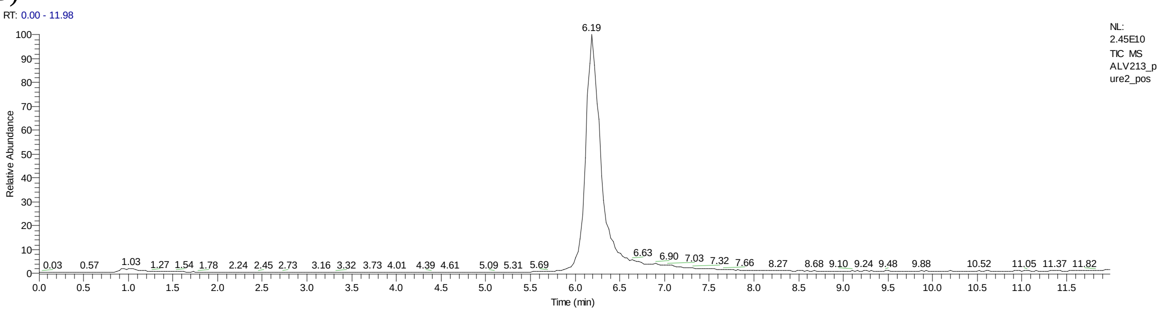

C)

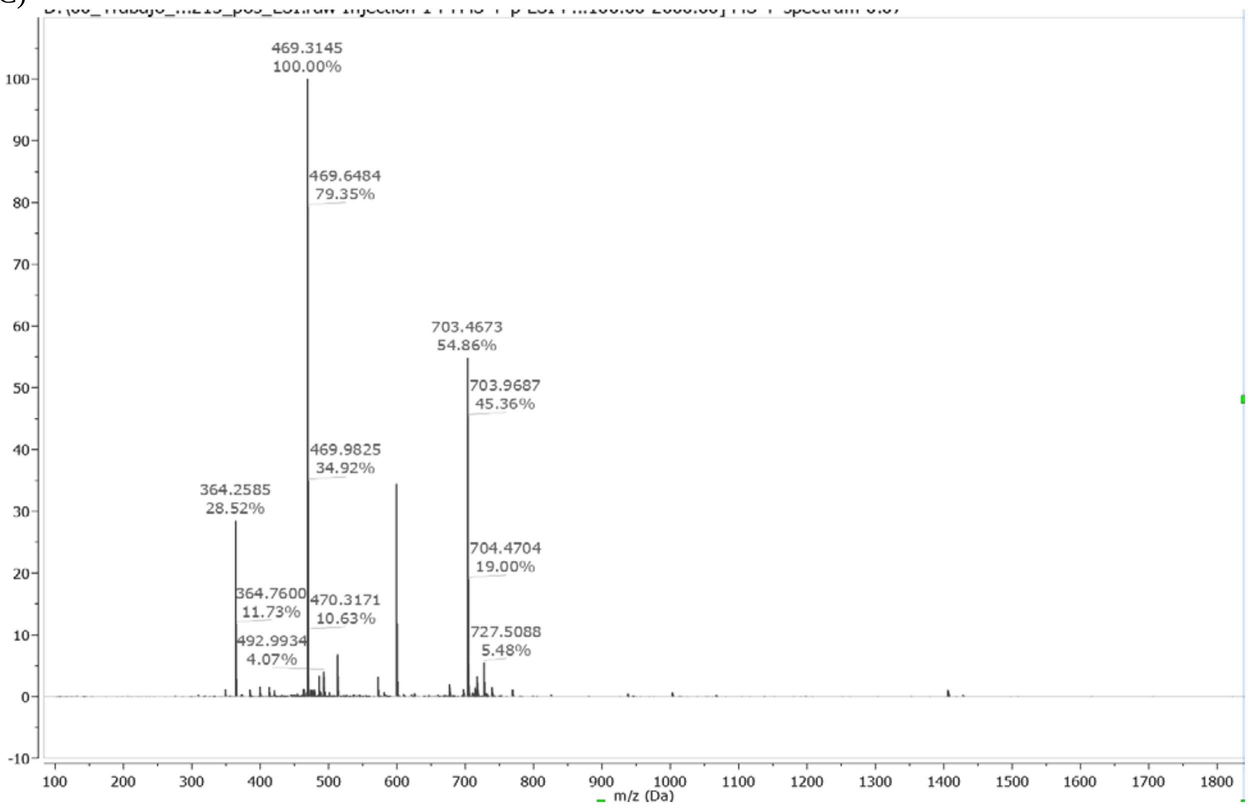

Figure S2. A) Structure, B) UHPLC-MS trace and C) ESI-HRMS spectrum of pure peptide **2**

A)

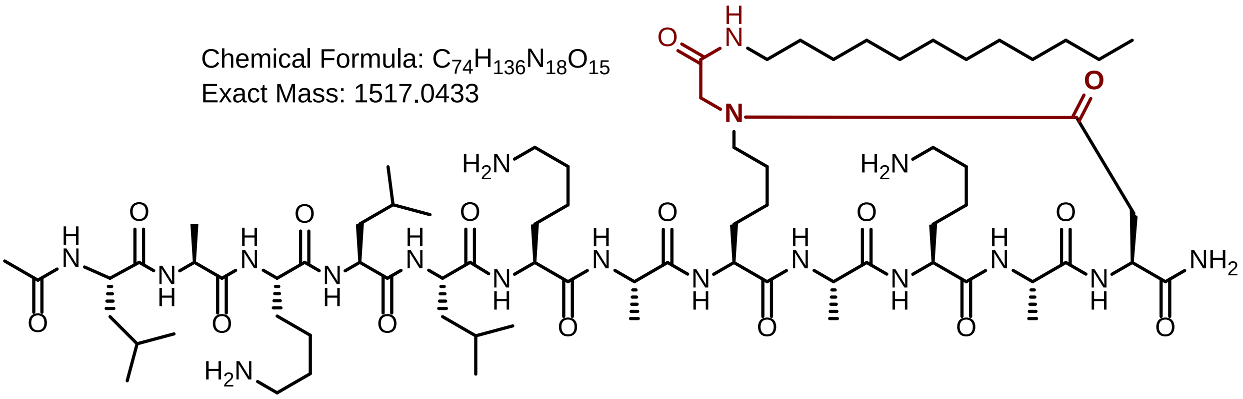

B)

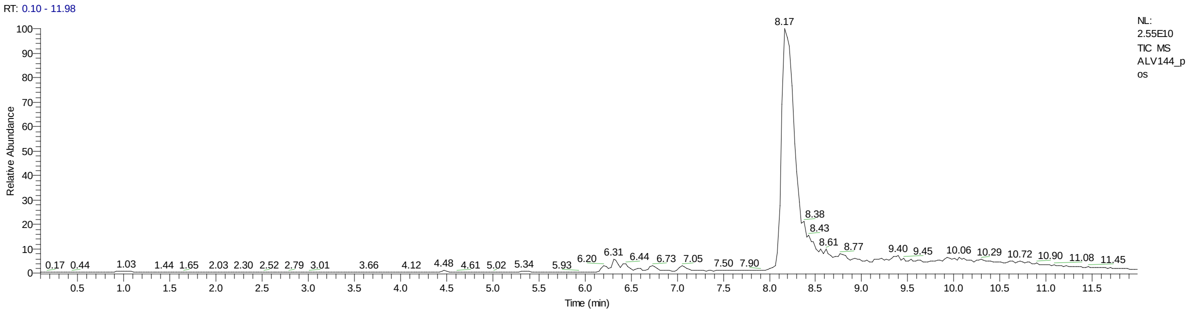

C)

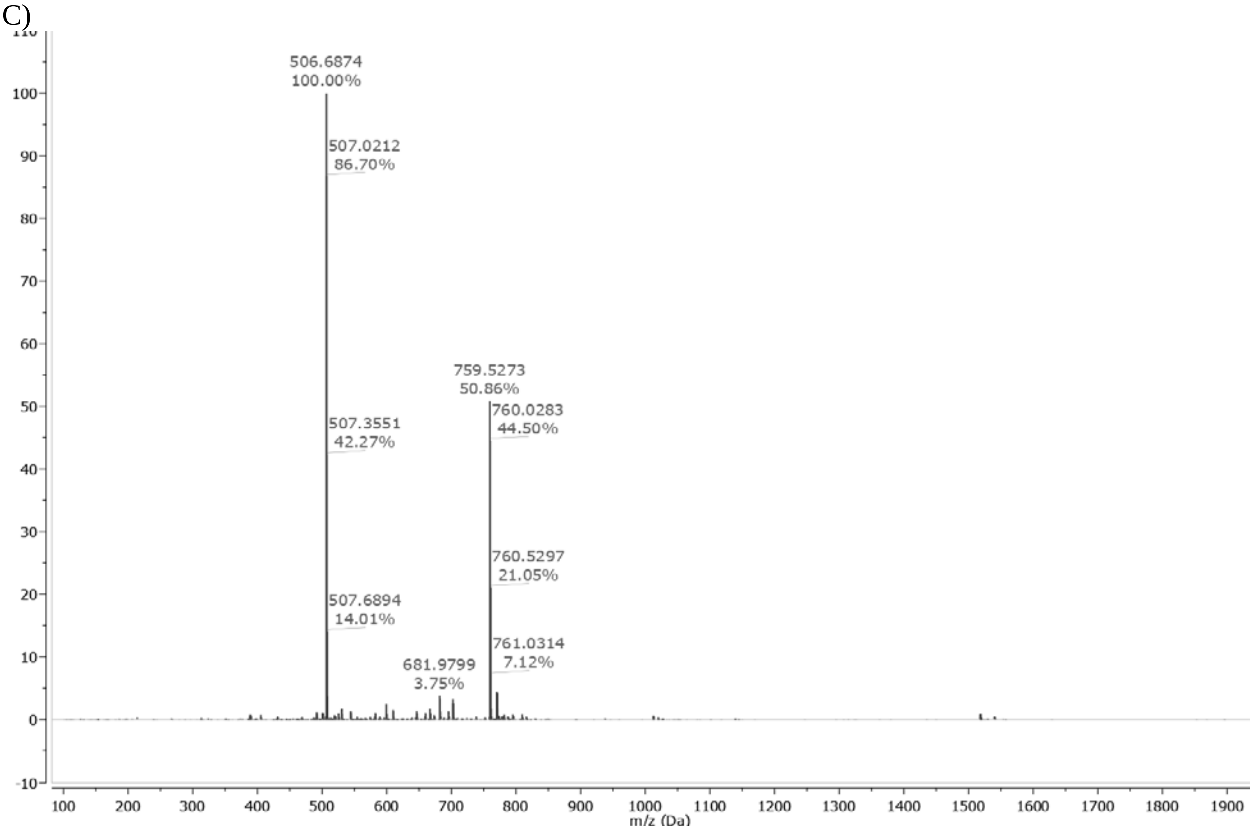

Figure S3. A) Structure, B) UHPLC-MS trace and C) ESI-HRMS spectrum of pure peptide **3**

A)

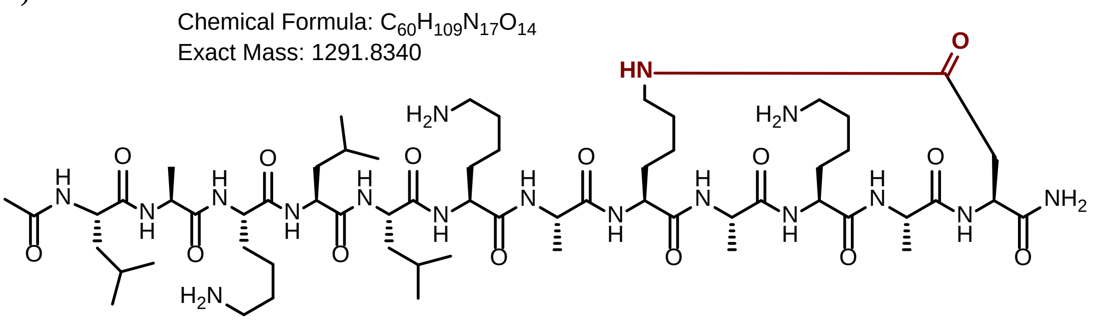

B)

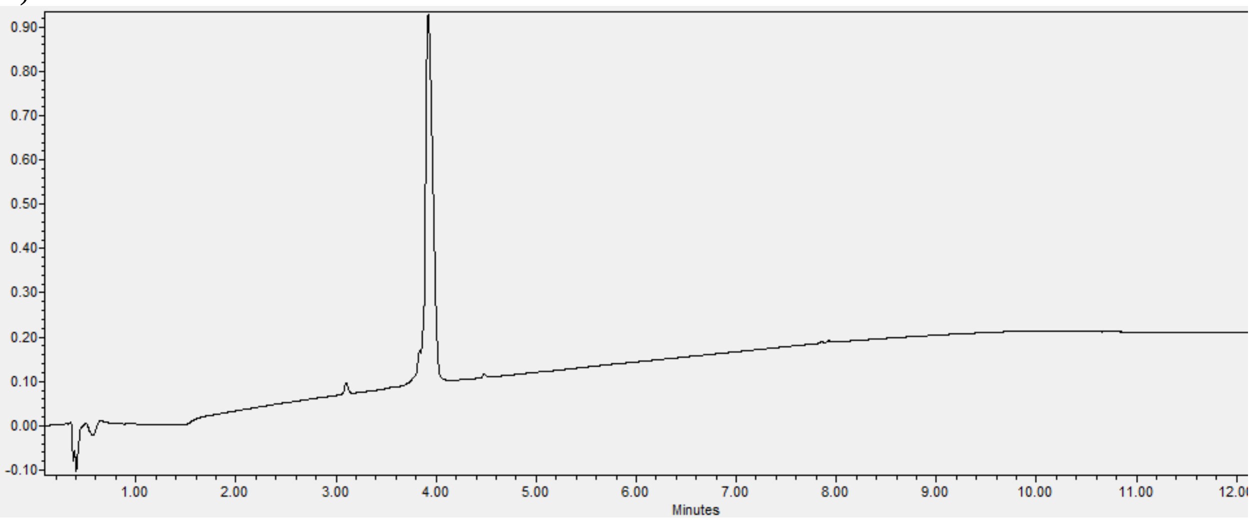

C)

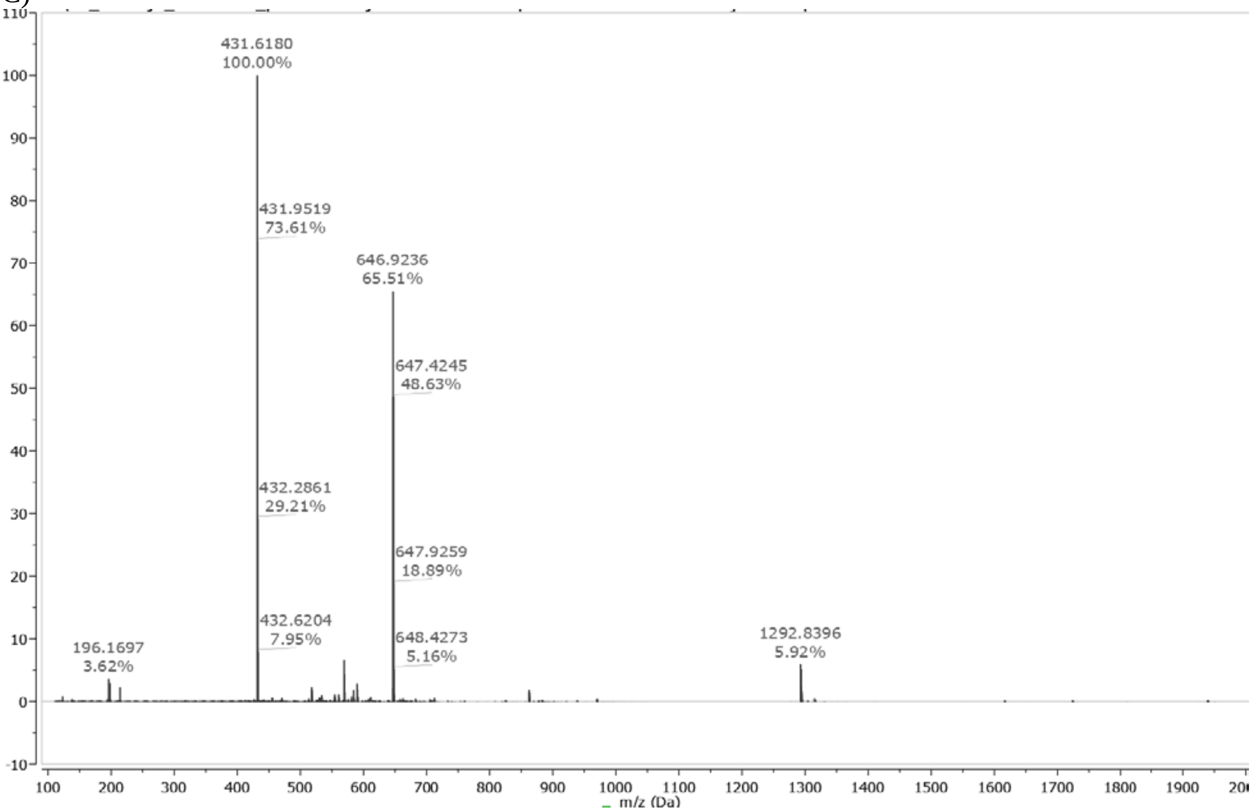

Figure S4. A) Structure, B) UHPLC trace at 200 nm and C) ESI-HRMS spectrum of pure peptide **4**

A)

B)

C)

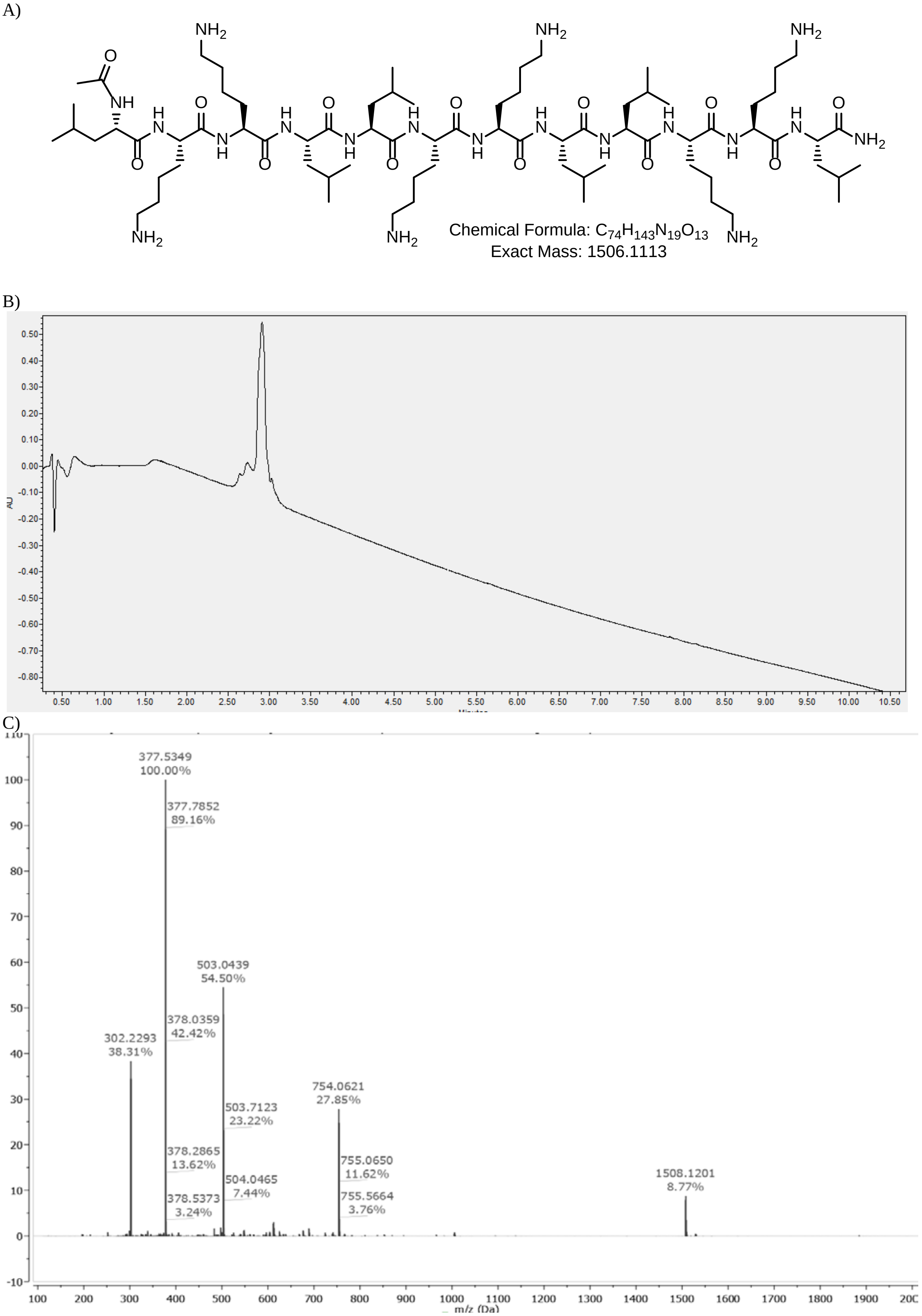

Figure S5. A) Structure, B) UHPLC trace at 200 nm and C) ESI-HRMS spectrum of crude peptide **5**

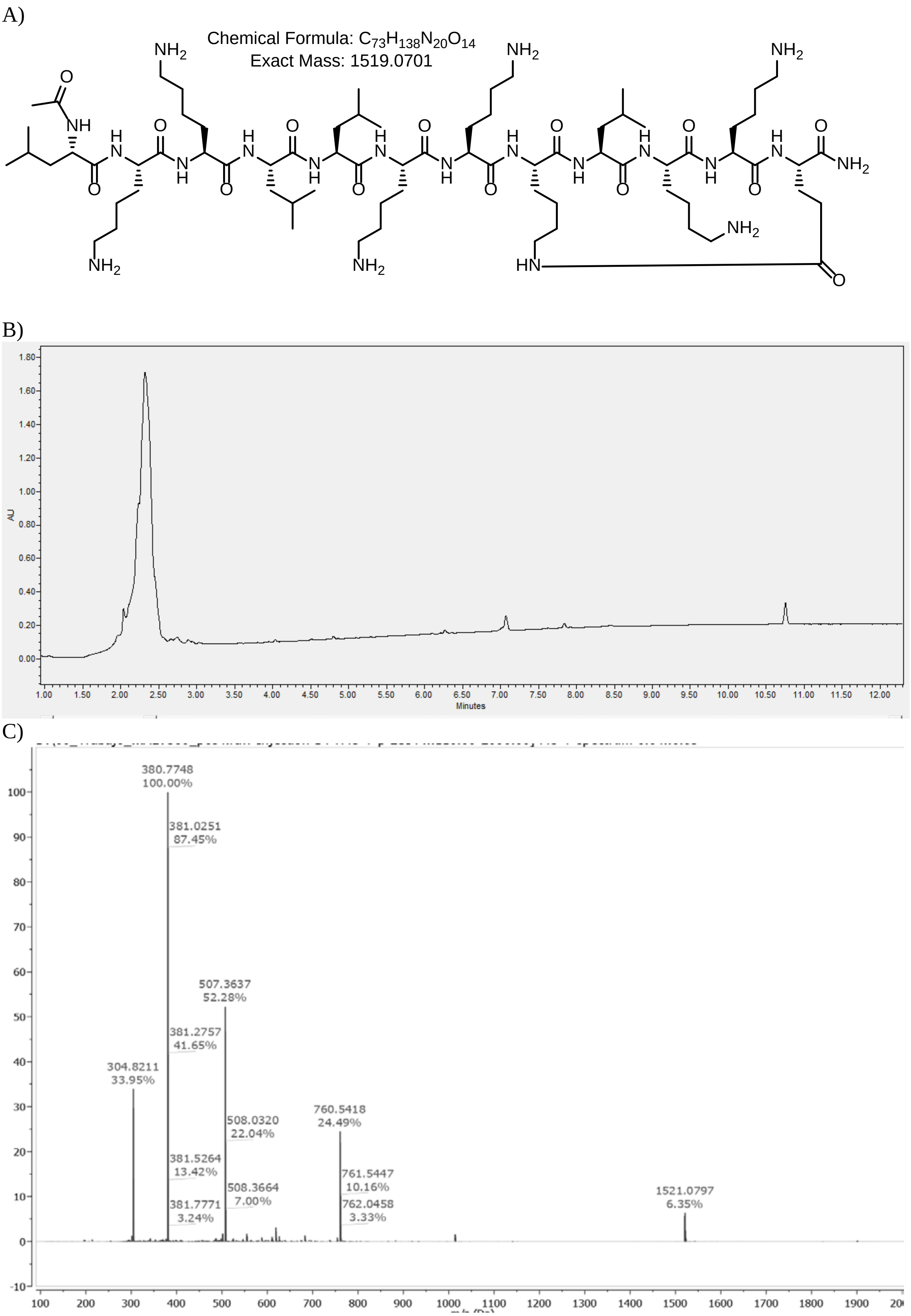

Figure S6. A) Structure, B) UHPLC trace at 200 nm and C) ESI-HRMS spectrum of pure peptide **6**

A)

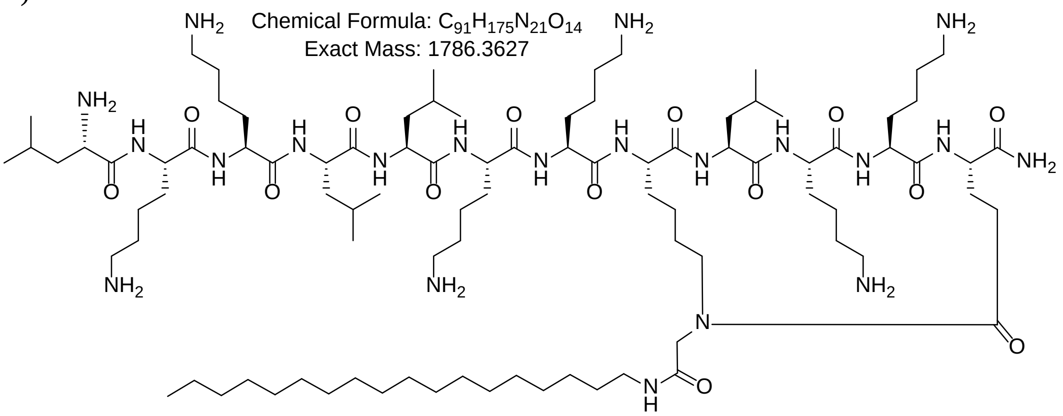

B)

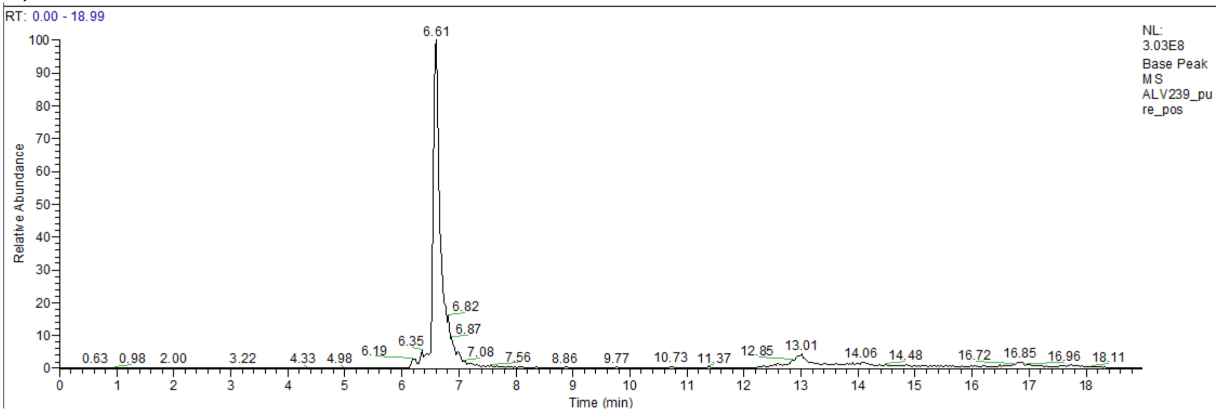

C)

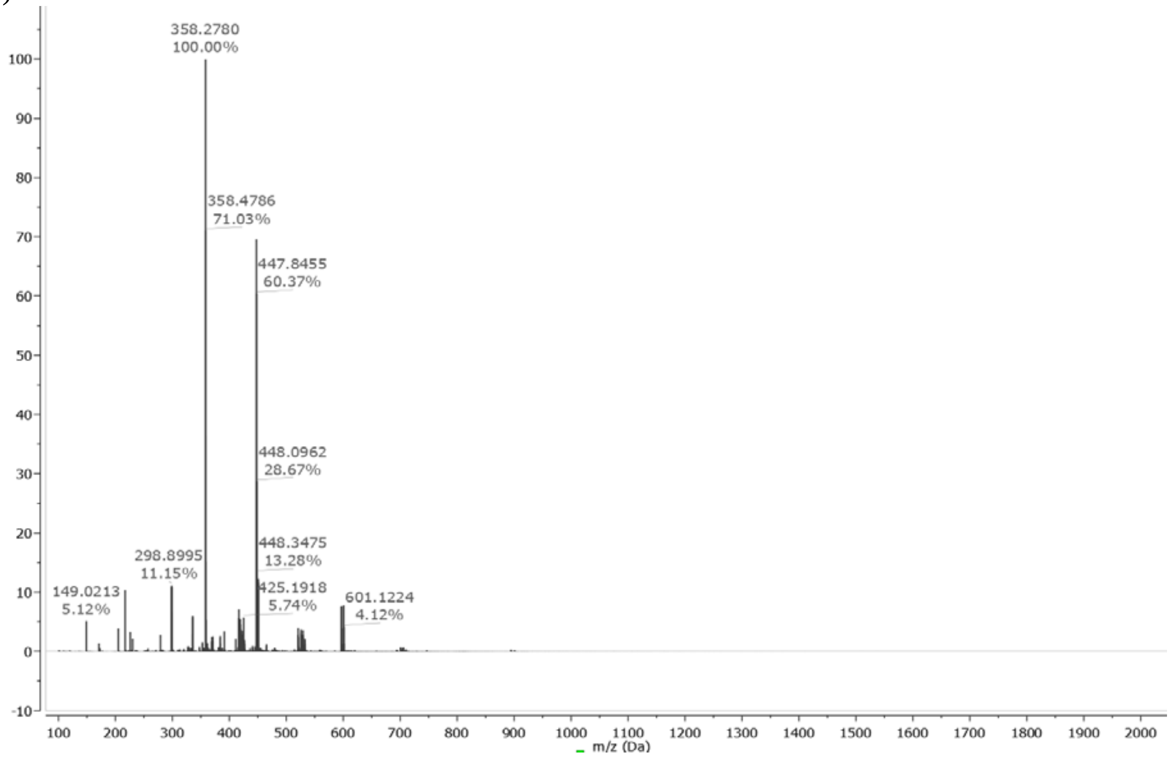

Figure S7. A) Structure, B) UHPLC-MS trace and C) ESI-HRMS spectrum of pure peptide **7**

A)

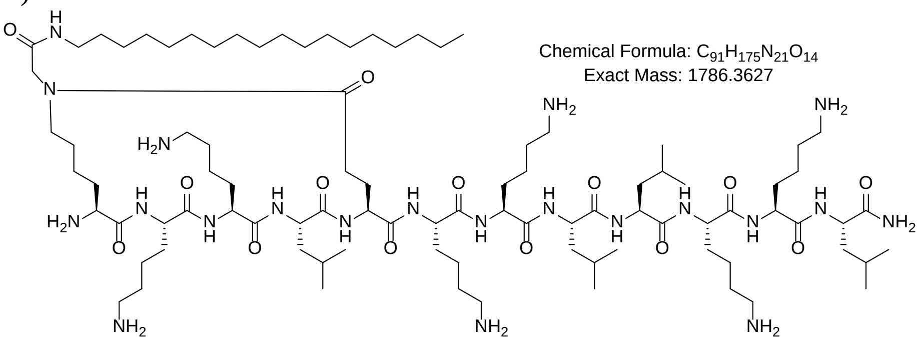

B)

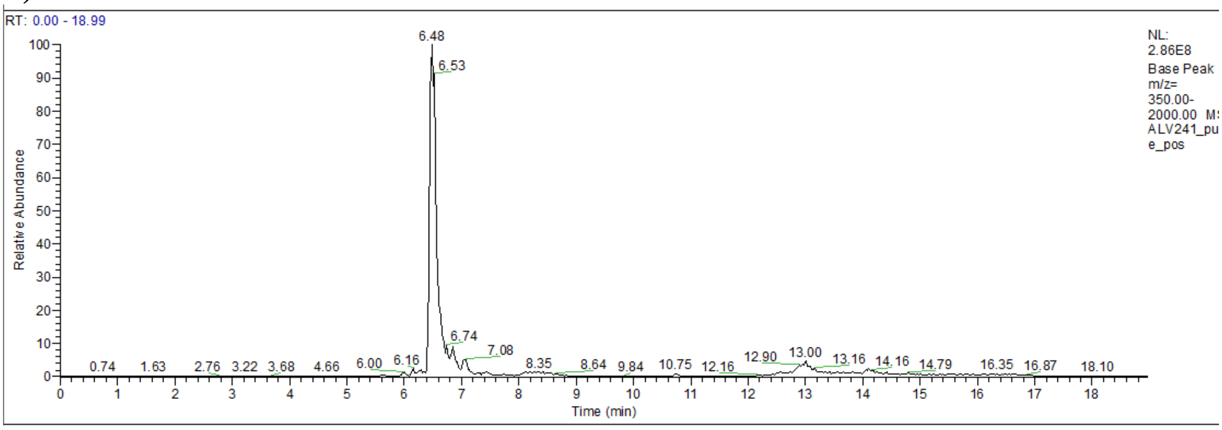

C)

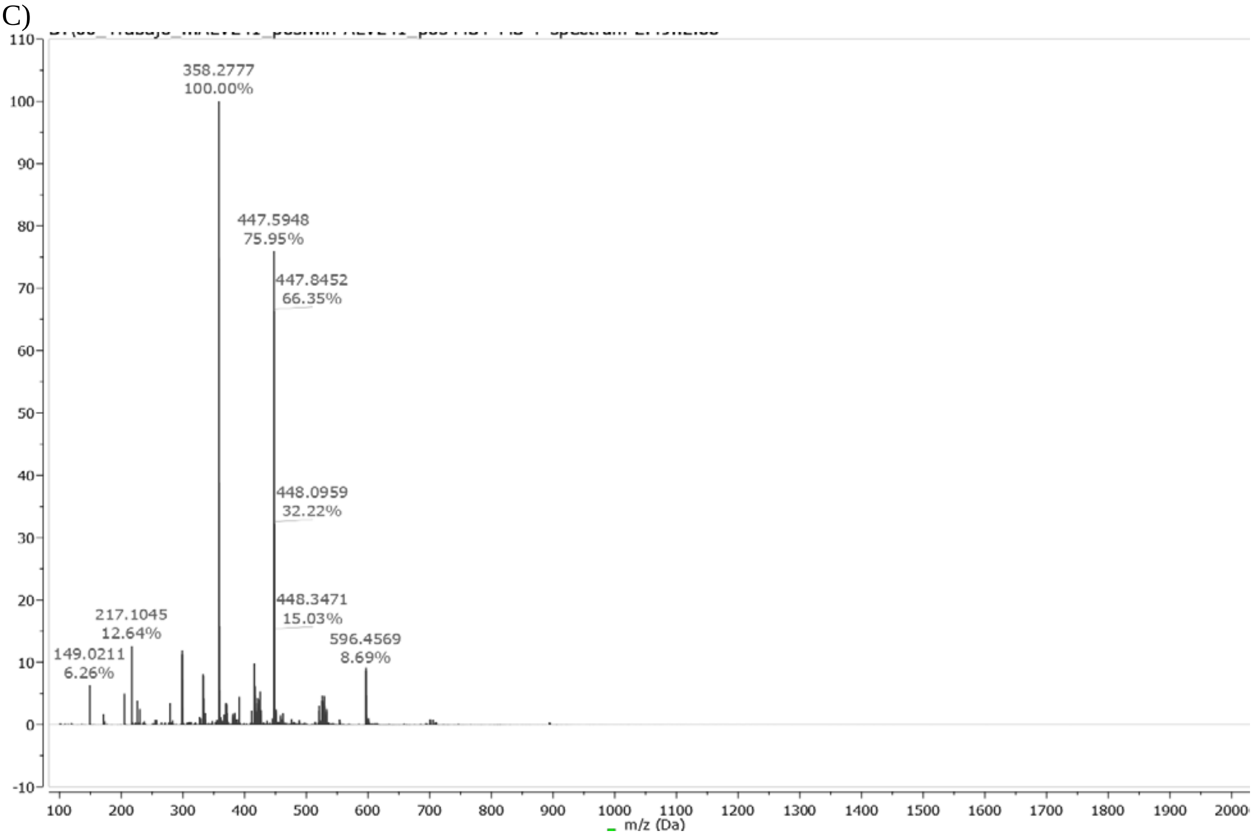

Figure S8. A) Structure, B) UHPLC-MS trace and C) ESI-HRMS spectrum of pure peptide **8**

A)

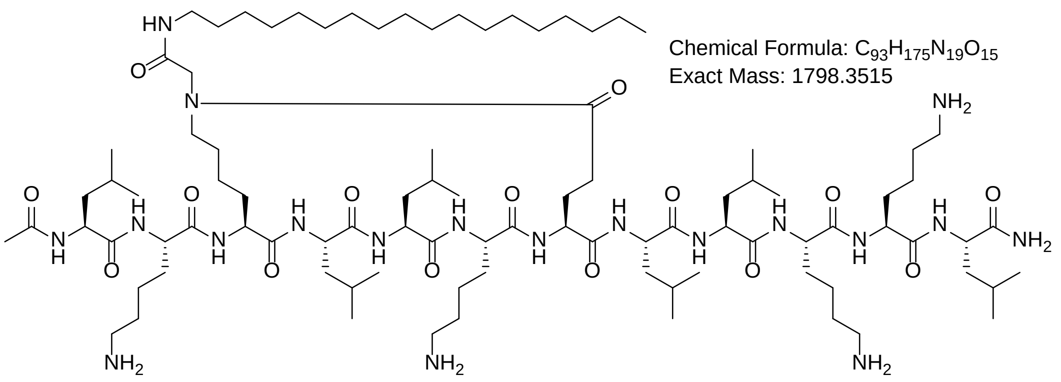

B)

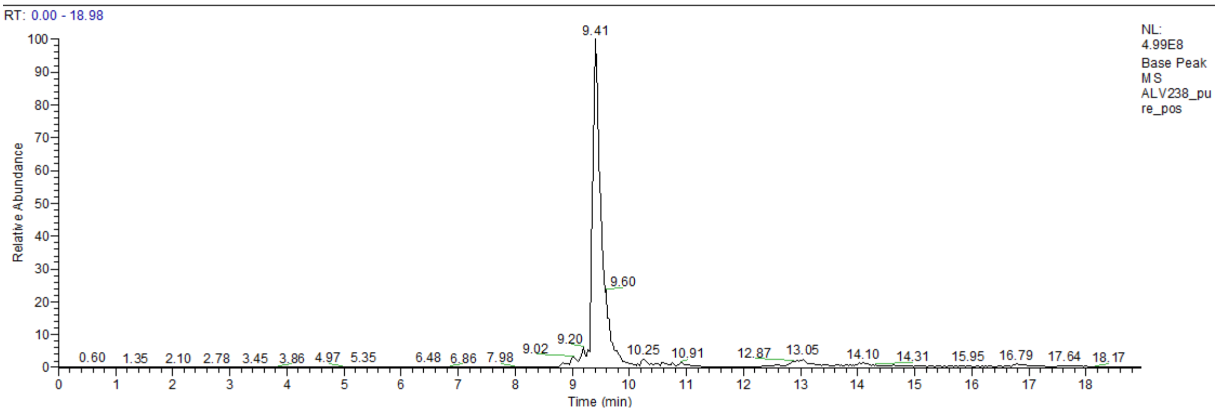

C)

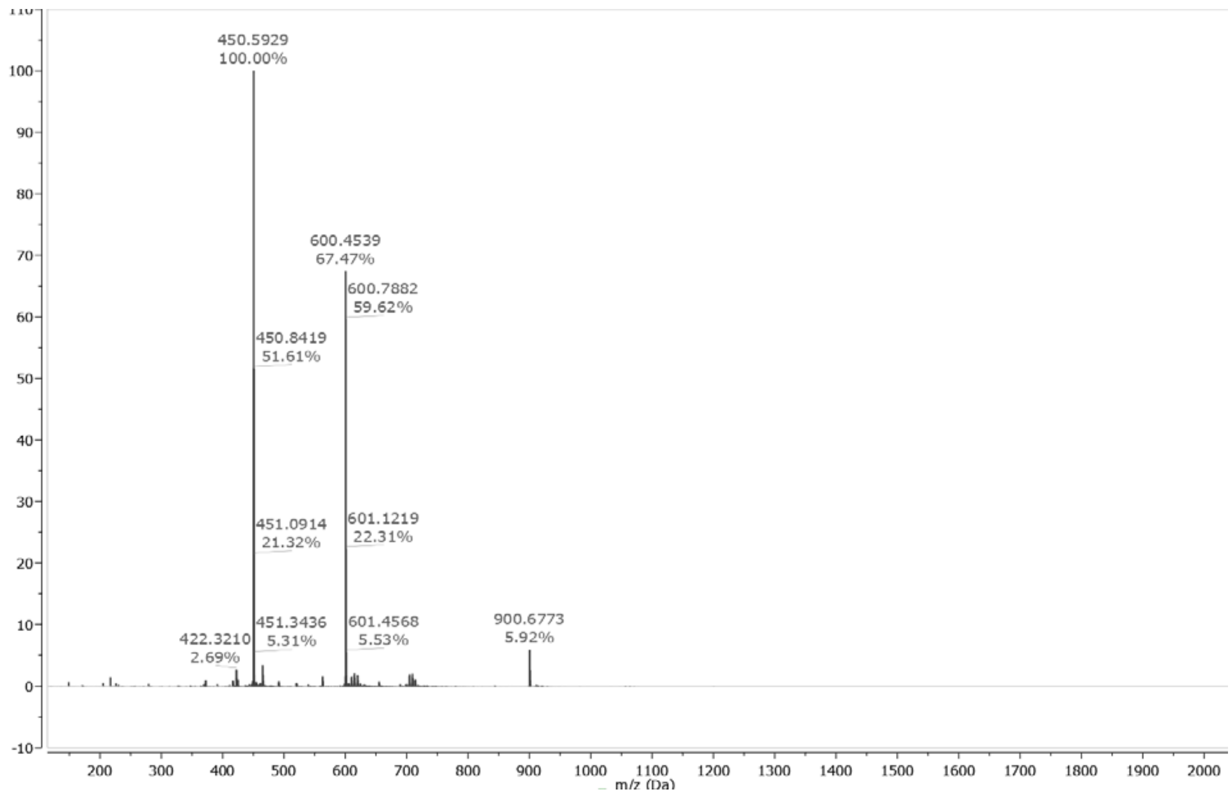

Figure S9. A) Structure, B) UHPLC-MS trace and C) ESI-HRMS spectrum of pure peptide **9**

A)

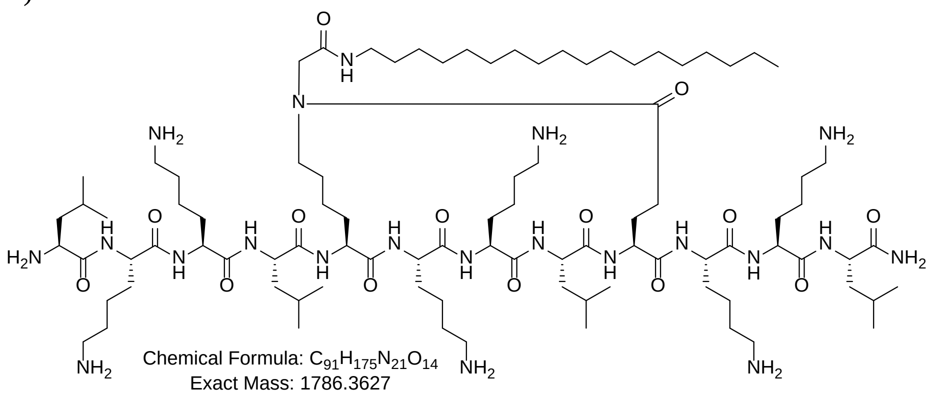

B)

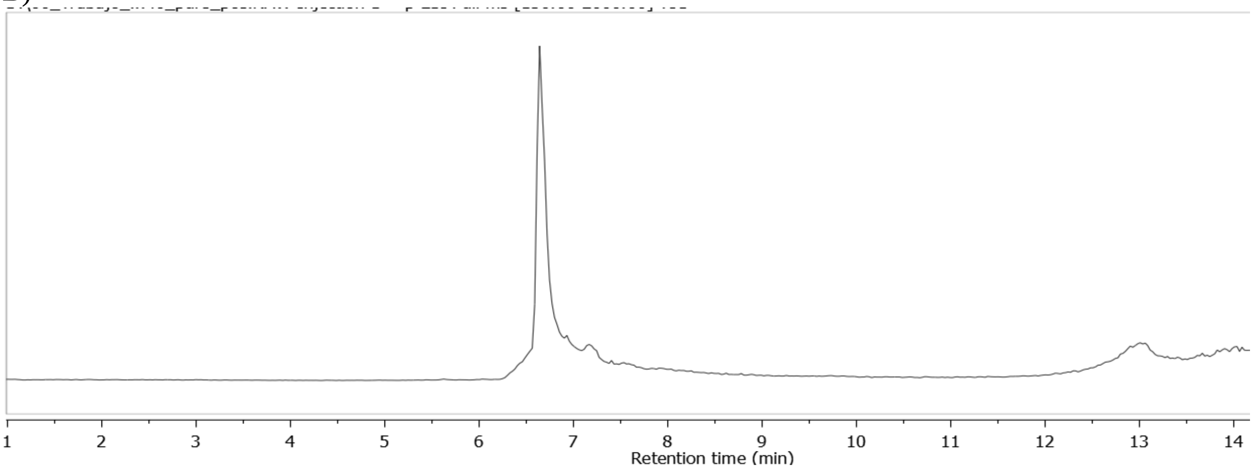

C)

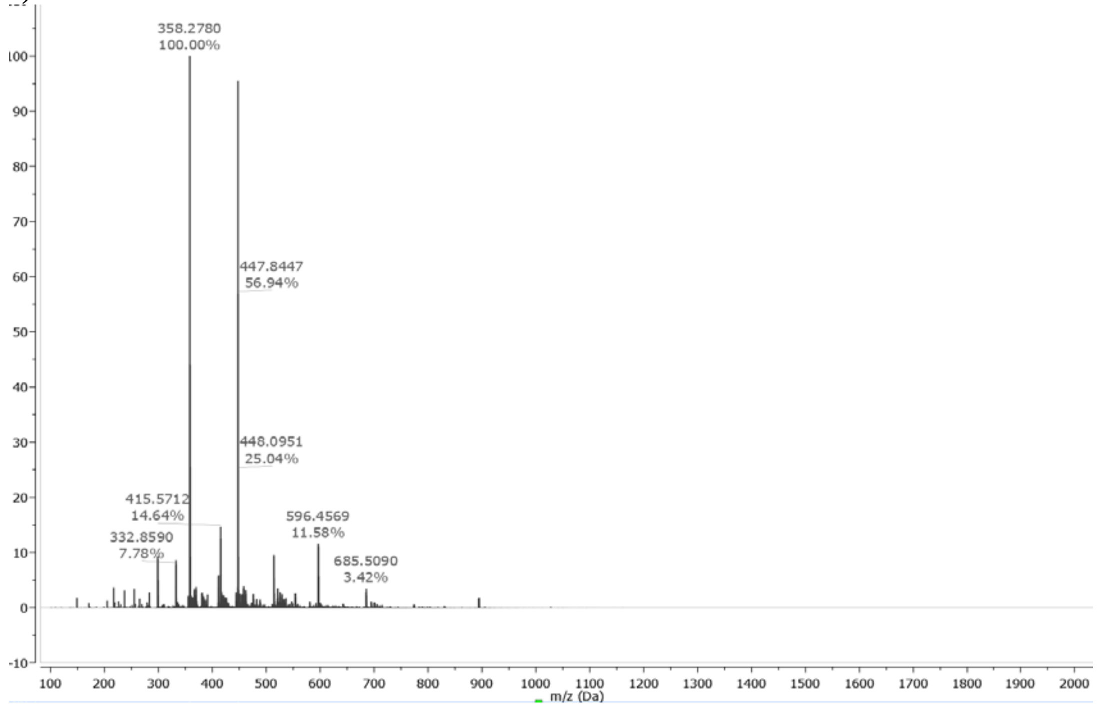

Figure S10. A) Structure, B) UHPLC-MS trace and C) ESI-HRMS spectrum of pure peptide **10**

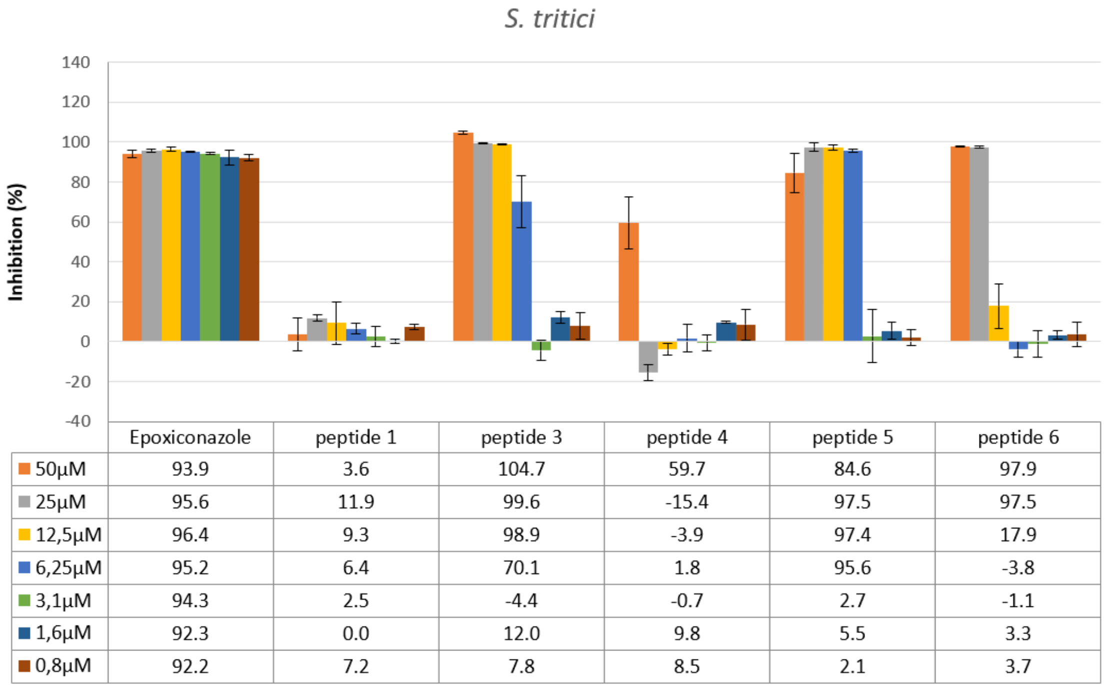

	Epoxiconazole	peptide 1	peptide 3	peptide 4	peptide 5	peptide 6
50µM	93.9	3.6	104.7	59.7	84.6	97.9
25µM	95.6	11.9	99.6	-15.4	97.5	97.5
12,5µM	96.4	9.3	98.9	-3.9	97.4	17.9
6,25µM	95.2	6.4	70.1	1.8	95.6	-3.8
3,1µM	94.3	2.5	-4.4	-0.7	2.7	-1.1
1,6µM	92.3	0.0	12.0	9.8	5.5	3.3
0,8µM	92.2	7.2	7.8	8.5	2.1	3.7

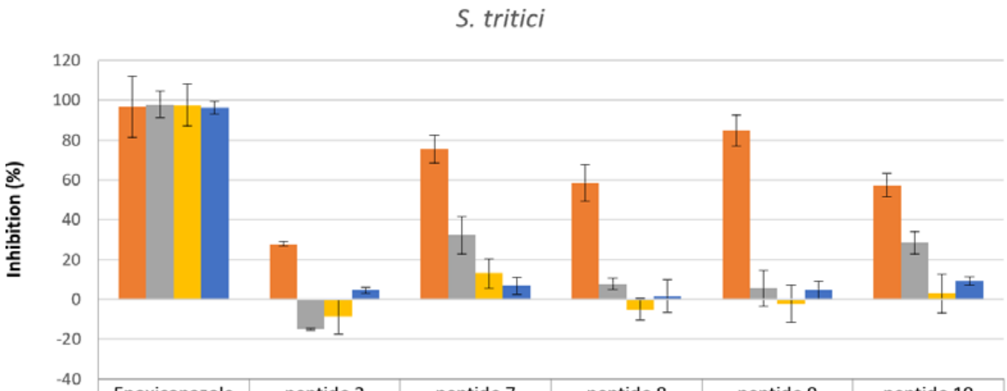

	Epoxiconazole	peptide 2	peptide 7	peptide 8	peptide 9	peptide 10
42 µM	96.60	27.80	75.53	58.51	84.85	57.52
14 µM	97.98	-14.86	32.24	7.98	5.76	28.49
5 µM	97.60	-8.50	13.16	-4.83	-2.22	3.07
1,5 µM	96.24	4.59	6.95	1.77	4.90	9.45

Figure S11. Inhibitory activity of the compounds against *Septoria tritici* pathogen.

Note: Inhibition values slightly above 100% as in peptide 3 could be the result of interference due to aggregation of the compound at relative high concentrations, resulting in low transmittance of the sample in the initial measurement (t_0).

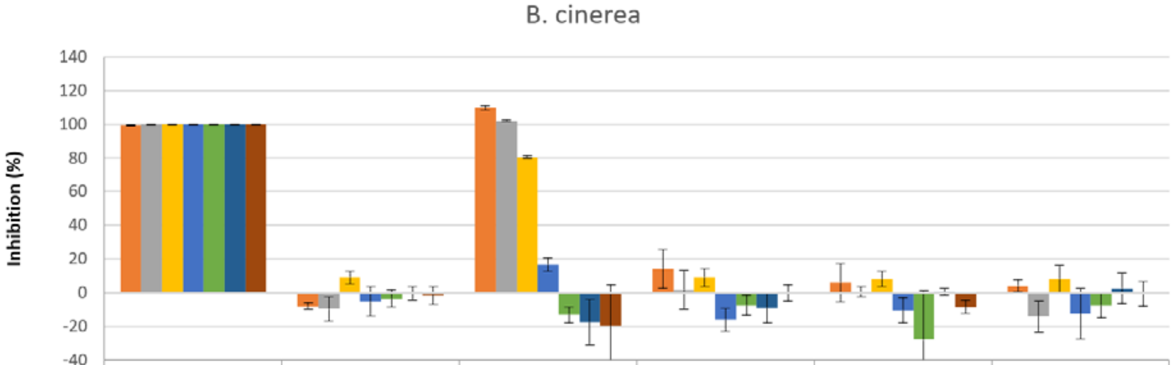

	Epoxiconazole	peptide 1	peptide 3	peptide 4	peptide 5	peptide 6
50µM	99.4	-7.9	109.7	14.2	6.0	4.1
25µM	99.6	-9.6	101.8	1.5	0.6	-13.9
12,5µM	99.7	9.0	80.4	9.1	8.1	8.0
6,25µM	99.8	-5.1	16.7	-16.0	-10.5	-12.5
3,1µM	99.6	-3.3	-13.0	-7.4	-27.6	-7.5
1,6µM	99.7	-0.4	-17.5	-8.9	0.7	2.7
0,8µM	99.7	-1.4	-19.8	-0.3	-8.5	-0.6

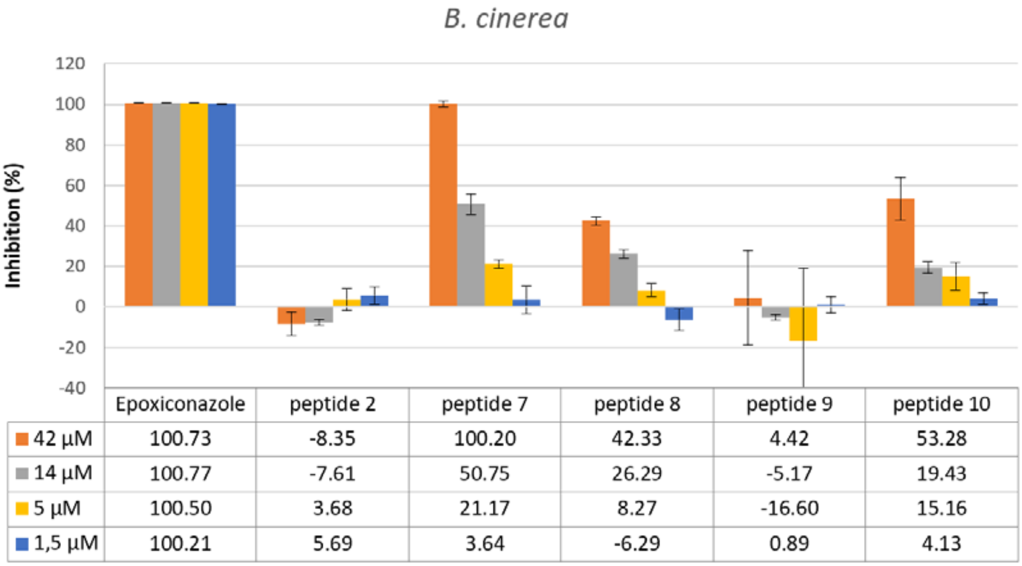

	Epoxiconazole	peptide 2	peptide 7	peptide 8	peptide 9	peptide 10
42 µM	100.73	-8.35	100.20	42.33	4.42	53.28
14 µM	100.77	-7.61	50.75	26.29	-5.17	19.43
5 µM	100.50	3.68	21.17	8.27	-16.60	15.16
1,5 µM	100.21	5.69	3.64	-6.29	0.89	4.13

Figure S12. Inhibitory activity of the compounds against *Botrytis cinerea* pathogen

Note: Inhibition values slightly above 100% as in peptide 3 could be the result of interference due to aggregation of the compound at relative high concentrations, resulting in low transmittance of the sample in the initial measurement (t_0).

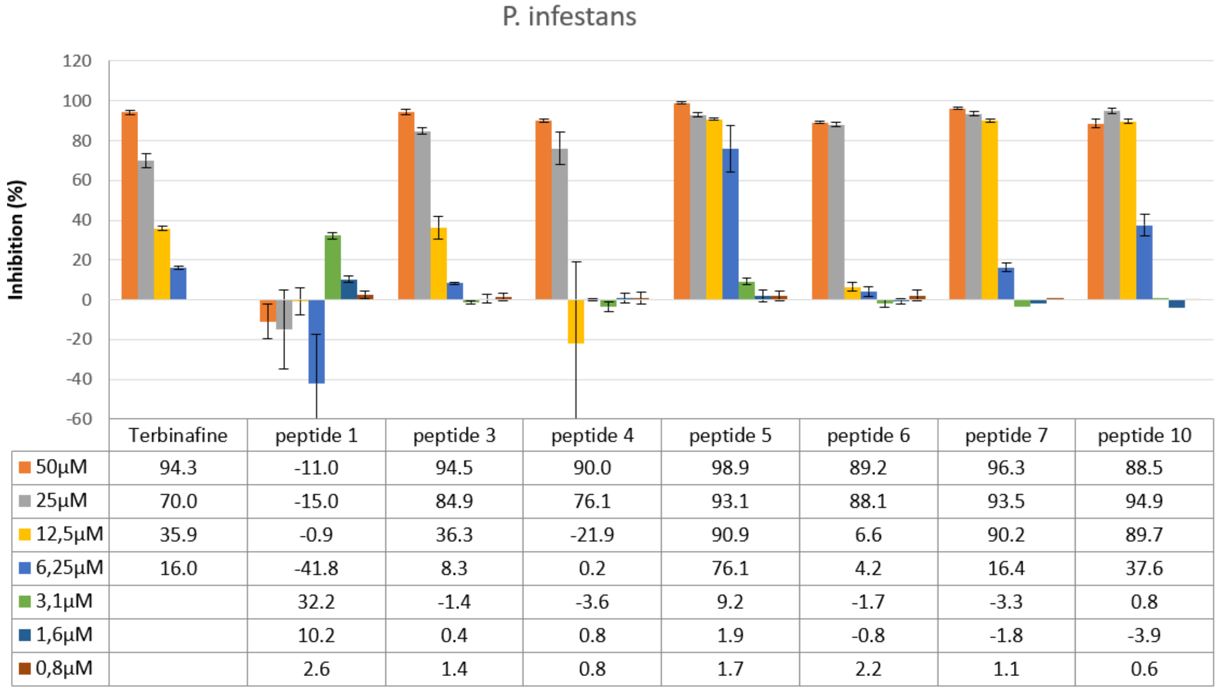

	Terbinafine	peptide 1	peptide 3	peptide 4	peptide 5	peptide 6	peptide 7	peptide 10
50μM	94.3	-11.0	94.5	90.0	98.9	89.2	96.3	88.5
25μM	70.0	-15.0	84.9	76.1	93.1	88.1	93.5	94.9
12,5μM	35.9	-0.9	36.3	-21.9	90.9	6.6	90.2	89.7
6,25μM	16.0	-41.8	8.3	0.2	76.1	4.2	16.4	37.6
3,1μM		32.2	-1.4	-3.6	9.2	-1.7	-3.3	0.8
1,6μM		10.2	0.4	0.8	1.9	-0.8	-1.8	-3.9
0,8μM		2.6	1.4	0.8	1.7	2.2	1.1	0.6

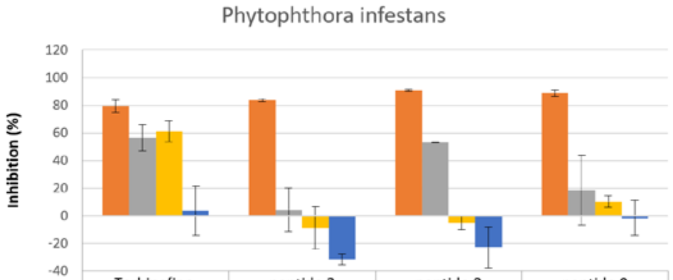

	Terbinafine	peptide 2	peptide 8	peptide 9
42 μM	79.34	83.52	90.75	88.80
14 μM	56.38	4.14	53.20	18.66
5 μM	61.07	-8.84	-5.12	10.17
1,5 μM	3.58	-31.40	-22.84	-1.69

Figure S13. Inhibitory activity of the compounds against *Phytophthora infestans* pathogen

Synthesis of Isocyanides

The non-commercial *n*-dodecyl isocyanide and *n*-octadecyl isocyanide were synthesized according to the protocols described in previous reports.[1]

[1] Pérez-Labrada, K.; Brouard, I.; Méndez, I.; Rivera, D.G. Multicomponent synthesis of Ugi-type ceramide analogues and neoglycolipids from lipidic isocyanides. *J. Org. Chem.* **2012**, *77*, 4660–4670.
